# Developmentally dynamic, non-convergent transcriptomic profiles in CNV models for schizophrenia risk

**DOI:** 10.1038/s41380-026-03679-0

**Published:** 2026-06-18

**Authors:** Hayley French, Shuyang Yao, Lisa Bast, Amparo Roig Adam, Paul Stümpges, Fatima Memic, Jennifer Mulle, Patrick F. Sullivan, Jens Hjerling-Leffler

**Affiliations:** 1 Division of Molecular Neurobiology, Department of Medical Biochemistry and Biophysics, Karolinska Institutet, Stockholm, Sweden; 2 Department of Medical Epidemiology and Biostatistics, Karolinska Institutet, Stockholm, Sweden; 3 Department of Functional Genomics, Center for Neurogenomics and Cognitive Research (CNCR), Vrije Universiteit Amsterdam, Amsterdam, The Netherlands; 4 Institute of Human Genetics, Heidelberg University, Heidelberg, Germany; 5 Department of Psychiatry, Robert Wood Johnson School of Medicine, Rutgers University, New Brunswick, New Jersey USA; 6 Center for Advanced Biotechnology and Medicine, Rutgers University, Piscataway, New Jersey USA; 7 Departments of Genetics, University of North Carolina at Chapel Hill, Chapel Hill, North Carolina USA; 8 Departments of Psychiatry, University of North Carolina at Chapel Hill, Chapel Hill, North Carolina USA; 9 Present Address: Tumor Biology and Therapeutics, Sprint Bioscience AB, Huddinge, Sweden

**Keywords:** Genetics, Molecular biology, Schizophrenia

## Abstract

Schizophrenia is a complex psychiatric disorder with significant genetic and clinical heterogeneity. Although numerous rare copy number variations (CNVs) with high risk for schizophrenia have been identified, they show no obvious overlap in gene content or function. We hypothesized that the downstream effects of schizophrenia-associated CNVs converge on shared molecular pathways. To test this, we profiled the prefrontal cortex of five schizophrenia-associated CNV mouse models — 15q13.3del, 3q29del, 1q21.1del, 22q11.2del, and 16p11.2dup — using single-cell RNA sequencing across two developmental stages: adolescence and adulthood. From 292,943 high-quality single-cell transcriptomes, we identified distinct age- and cell type-specific patterns of differential gene expression and biological pathway perturbations in each model. Rather than converging on a shared molecular mechanism, each CNV affected unique cellular pathways in a developmentally dynamic manner. Notably, genes dysregulated in deep-layer corticothalamic projection neurons from 15q13.3del and 16p11.2dup models, and intratelencephalic neurons from adult 22q11.2del mice, showed enrichment for schizophrenia-SNP heritability. These results support a model in which rare CNVs contribute to schizophrenia genetic risk through developmentally dynamic, distinct pathways rather than through a shared molecular mechanism.

## Introduction

Schizophrenia is a complex psychiatric disorder characterized by profound disruptions in cognition, perception, and behavior, affecting millions globally [1]. The transition from adolescence to adulthood represents a critical developmental window during which the first symptoms of schizophrenia often manifest [2], making it particularly relevant for studying the neurobiological consequences of genetic risk. Major efforts from genomic studies have helped elucidate the genetic architecture of schizophrenia, which is now understood to be highly polygenic, in that combinations of common single-nucleotide polymorphisms (SNPs), rare copy number variations (CNVs), and rare single-nucleotide variants (SNVs) collectively contribute to the risk of developing the disorder [3–8]. While our understanding of the genetic basis of schizophrenia has advanced considerably, there remains a disconnect between our understanding of genetic risk and disease biology.

Bridging this gap requires experimental approaches that integrate genomic findings with neurobiological readouts [6]. Since ~287 schizophrenia loci of individually small effect have been identified [8], the potential for modelling common variant risk in mice remains challenging. Rare variants, including CNVs, provide a more promising avenue for modelling genetic risk due to high penetrance and strong associations with schizophrenia (odds ratios range from around 5–60, unlike SNPs whose maximum effects are usually < 1.25) [5, 6]. Importantly, in the case of 22q11.2 deletion syndrome, the major phenotypic features of schizophrenia, including the mean age of psychosis onset and disease course, do not differ from those in individuals with schizophrenia who do not carry a CNV [9]. This suggests that despite their rarity, CNVs could offer an informative window into mechanisms that may be broadly relevant to idiopathic schizophrenia.

Given that less than 2% of schizophrenia cases carry a schizophrenia-associated CNV [3], human post-mortem brain samples are rare. For this reason, experiments studying CNVs are typically conducted using cell or animal models. Recent studies have made significant advances by generating neurons and brain organoids from induced pluripotent stem cells (iPSCs) derived from individuals with CNV syndromes. These studies have found critical changes in neuronal function, such as neurotransmitter release, calcium signaling, and neuronal activity, along with alterations in the expression of genes associated with schizophrenia [10–14]. At least six schizophrenia-associated CNVs—1q21.1 deletion, *NRXN1* deletion, 15q13.3 deletion, 16p11.2 duplication, 22q11.2 deletion, and 3q29 deletion—have been modelled in mice and are reported to exhibit varying degrees of schizophrenia-relevant phenotypes [15–36]. Beyond schizophrenia, these mouse lines also model the broader clinical heterogeneity of CNV syndromes, which includes neurodevelopmental disorders that manifest early, like attention deficit/hyperactivity disorder (ADHD), autism spectrum disorder (ASD), developmental delay (DD), and intellectual disability (ID), along with physical abnormalities such as dysmorphic facial features and congenital heart disease [37, 38]. By capturing this clinical variability, cell and animal models represent valuable tools for understanding the biology of rare CNV syndromes.

Single-cell technologies have emerged as a crucial readout for large-scale molecular investigations at the resolution of individual cells, building on bulk tissue analyses by resolving transcriptional changes across the brain’s diverse cell types [39, 40]. In this study, we used single-cell RNA-sequencing (scRNA-seq) to explore potential molecular convergence across five schizophrenia-associated CNVs in the mouse prefrontal cortex. We selected two key developmental time points, post-puberal adolescence and early adulthood, to align with the period when schizophrenia symptoms typically emerge in humans. Transcriptomic analysis between these ages on bulk brain tissue has suggested that gene networks involved in neuronal energetics might be commonly perturbed in three CNV mouse models (15q13.3del, 22q11.2del and 1q21.1del) [41]. We hypothesized that shared molecular mechanisms may underlie schizophrenia risk during this developmental window. Our results reveal that rare schizophrenia-associated CNVs produce distinct age- and cell type-specific transcriptomic effects, with minimal overlap in gene expression or pathways. These findings suggest that the shared risk for schizophrenia may arise from convergence at a biological level beyond transcriptomics.

## Results

**Fig. 1:**
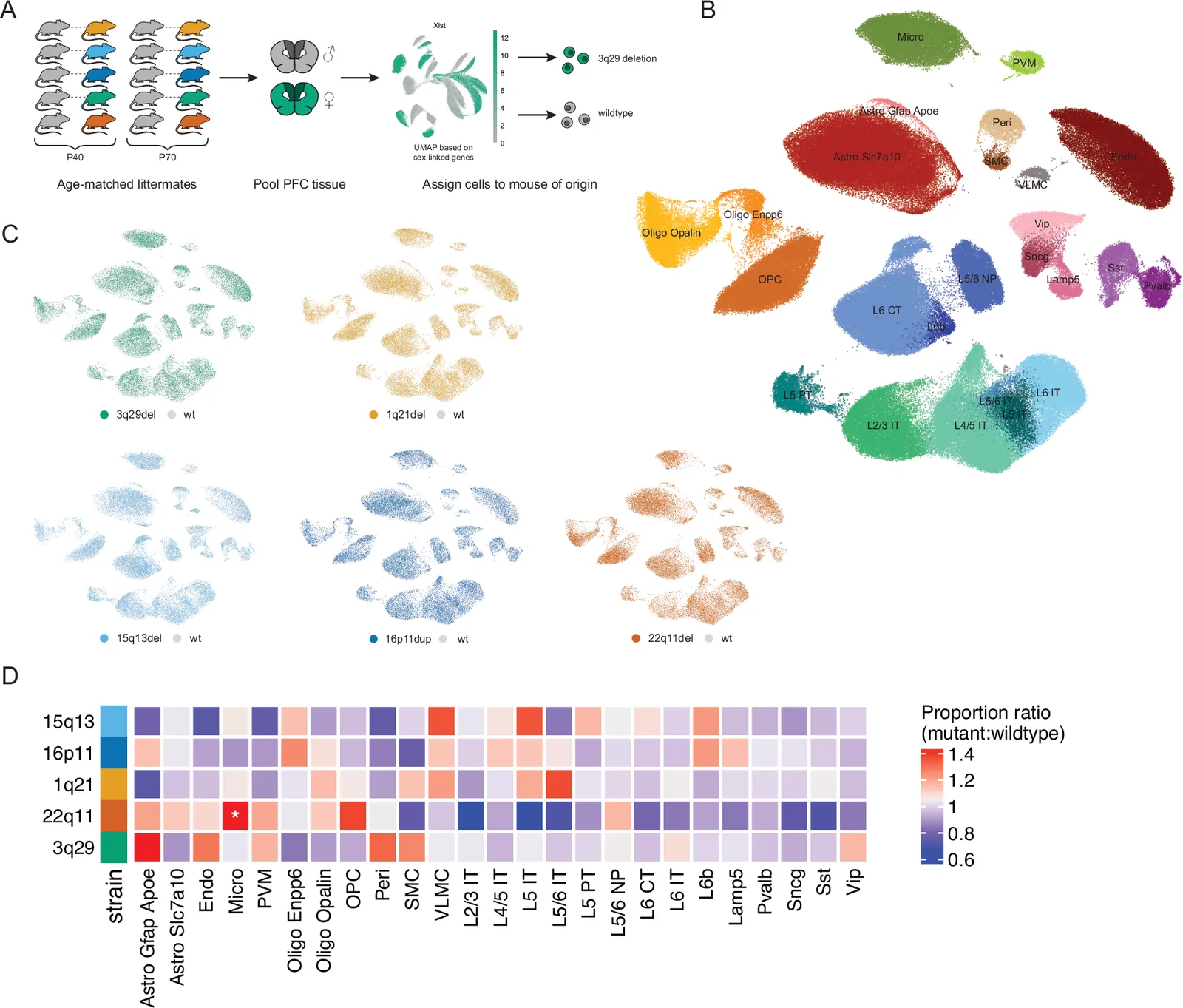
Experimental workflow and overview of the dataset. **A** Schematic of scRNA-seq data generation with paired experimental design. **B** UMAP representation of the RNA-expression profiles of 292,943 single cells analyzed from 80 mice, colored by cell type annotation. Abbreviations: Astro, astrocyte; endo, endothelial cell; oligo, oligodendrocyte; OPC, oligodendrocyte precursor cell; peri, pericyte; PVM, perivascular macrophage; SMC, smooth muscle cell; VLMC, vascular leptomeningeal cells; IT, intratelencephalic; PT, pyramidal tract; NP, near-projecting; CT, corticothalamic. **C** UMAP representations of the RNA-expression profiles from CNV mutant mice and their wildtype littermates, separated by CNV model and colored by genotype. **D** Ratio of cell type proportions between mutant and wildtype mice, grouped by CNV model. Red indicates a higher proportion in mutants, while blue indicates a higher proportion in wildtype mice. Asterisks indicate significant differences (FDR < 0.05).

To explore molecular convergence across distinct genetic risk factors for schizophrenia, we performed whole-cell RNA sequencing of the prefrontal cortex in five hemizygous mouse models of human CNVs, all maintained on the same background strain (C57BL/6 N): 15q13.3del, 3q29del, 1q21.1del, 22q11.2del, and 16p11.2dup (Supplementary Table 1). Tissue was collected at postnatal days 40 (P40, post-puberal adolescence) and 70 (P70, early adulthood) [42]. In total, 80 mice were included: for each CNV, 8 mutants (4 per age group) and 8 matched controls (4 per age group). To minimize batch effects from sample processing and library preparation, we implemented a paired experimental design, where each mutant mouse was matched to a littermate control of the opposite sex, apart from the 22q11.2del model (Supplementary Note 1). Following the dissection of the prefrontal cortex, tissue from each control/mutant mouse pair was pooled together and processed as a single sample. After sequencing, individual cells could be mapped back to the donor mouse and respective genotype based on the expression of *Xist*, a non-coding RNA that plays an important role in X chromosome inactivation in female mammals (Fig. 1A) [43].

Following stringent quality control, our dataset contained 292,943 high-quality single-cell transcriptomes (Supplementary Fig. 1). The mean number of genes detected per cell was 4477 and the mean number of cells captured per mouse was 3662. Twenty-five major cell types with distinct expression profiles were identified in all models (Fig. 1B, C, Supplementary Fig. 2). Non-neuronal cells were the predominant cell class (11 cell types, 51% of all cells), followed by excitatory projection neurons (9 cell types, 42% of all cells, 86% of neurons). Inhibitory interneurons were the smallest cell class (5 cell types, 7% of all cells, 14% of neurons). These proportions are in accordance with the most comprehensive whole mouse brain cell atlas to date, which reported 48% non-neuronal cells, 45% glutamatergic neurons, and 7% GABAergic neurons in the adult mouse isocortex [44].

We first assessed possible differences in cell type proportions between groups [45], finding a significantly increased proportion of microglia in 22q11.2del mice, while cell type composition remained unchanged in all other CNV models (Fig. 1D). When analyzing the adolescent and adult datasets separately, no significant changes were observed in adolescent mice, whereas a significant increase in the proportion of oligodendrocyte precursor cells was found in adult 22q11.2del mice (Supplementary Fig. 3A-B). Notably, we found no shared patterns of cell abundance changes across CNV models, suggesting that altered cell type proportions in the prefrontal cortex are unlikely to underlie their convergent risk for schizophrenia.

### Differential gene expression across models

**Fig. 2:**
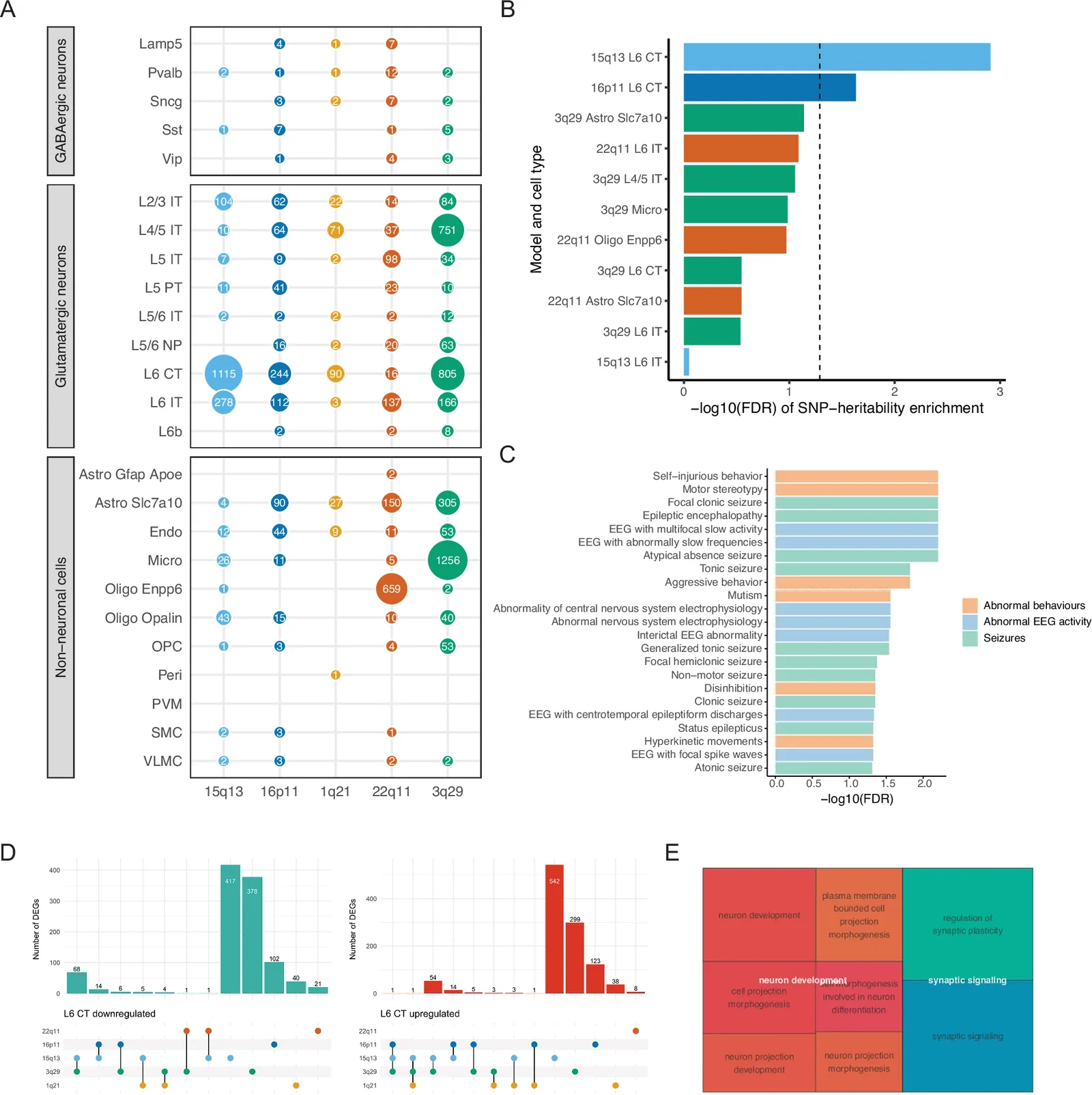
Glutamatergic neurons are broadly affected across CNV models, with L6 corticothalamic neurons implicated in schizophrenia genetic risk and disease-relevant pathways. **A** Total number of significant DEGs (FDR < 0.1), excluding genes in the CNV regions, identified in each cell type when comparing mutant mice to wildtype mice. **B** Significance of SNP-heritability enrichment in DEG lists. Only DEG lists that contain sufficient numbers of SNPs ( ≥ 1%) to yield robust estimates are shown. **C** Human pathology ontology terms enriched in L6 CT neuron DEGs from 15q13.3del mice, colored by broader classification categories. **D** Upset plots showing overlap of down- and upregulated DEGs from L6 CT neurons among all CNV models. **E** GO terms associated with the 124 L6 CT neuron DEGs shared between 15q13.3del and 3q29del, reduced based on their semantic similarity.

After carefully evaluating several methods using the detection of CNV locus genes as an internal control, we implemented a negative binomial generalized linear mixed model to fully leverage the pair-wise experimental design and identify differentially expressed genes (DEGs) between mutant and wildtype mice [46, 47]. Recognizing the increased power of a larger combined dataset, we first analyzed adolescent and adult timepoints jointly to characterize overall patterns across CNV models. As expected, in all CNV mouse models, most transcripts within the CNV loci showed reduced expression in the deletion models and increased expression in the duplication model (Supplementary Fig. 3C-D). No enrichment of gene expression changes was detected in genes located within 1 megabase outside of the CNV regions, indicating that the deletion and duplication events are localized to specific regions without cis-effects. Based on the numbers of DEGs, each of the five CNVs appeared to impact gene expression in a unique and cell type-specific manner (Fig. 2A). In general, glutamatergic neurons were the most affected cell class, exhibiting DEGs across many subtypes in most or all CNV models. We detected few DEGs in smaller cell populations (e.g., GABAergic neurons and many non-neuronal cell populations), likely due in part to reduced statistical power. Indeed, we observed moderate correlations (r = 0.49–0.60) between the number of cells analyzed and the number of DEGs detected across cell types in all models except 22q11.2del (Supplementary Fig. 3E). Nonetheless, the differential expression of several CNV locus genes in these sparse cell types suggests that meaningful biological effects can still be detected despite limited cell numbers (Supplementary Fig. 3D***)***.

Interestingly, in three CNV models (1q21.1del, 15q13.3del, and 16p11.2dup), layer 6 corticothalamic projection neurons had more DEGs than expected given the cluster size. Microglia and differentiation-committed oligodendrocyte precursors (Oligo Enpp6) were strongly and uniquely perturbed in 3q29del and 22q11.2del mice, respectively, highlighting model-specific effects. Detailed, model-specific results are presented in Supplementary Figs. 4–8*and* Supplementary Data File 1.

### Crosstalk between CNV loci, rare and common variants

Recognizing that schizophrenia is a complex genetic disorder involving several types of risk variants, we aimed to explore the expression of risk-associated genes within CNV models. First, we assessed whether ‘crosstalk’ existed among the CNV loci by examining if CNV locus genes were differentially expressed across other CNV models (e.g., whether 3q29del mice showed DEGs in the 22q11.2 region). While some evidence of crosstalk was observed, the number of DEGs at each CNV locus was consistent with chance expectations (p = 0.37–0.87, hypergeometric tests; Supplementary Table 2, 3). We also investigated whether rare coding variants associated with risk for schizophrenia in humans (FDR  <  5%, n = 26 mouse homologs, e.g., *Gria3* and *Grin2a*) [5] were enriched among the DEGs. While several of the identified risk variants showed differential expression across various cell types, most notably in glutamatergic neurons, their enrichment did not exceed what might occur by chance (p = 0.37, hypergeometric test, Supplementary Fig. 9A). Nevertheless, certain findings stand out, such as the differential expression of genes involved in ion transport in L6 corticothalamic neurons, with *Gria3* from 15q13.3 del and *Cacna1g* from 16p11.2dup both showing notable changes in this neuronal population. Among the eight rare coding variant genes identified as DEGs in our dataset, four (*Hist1h1e*, *Trio*, *Cacna1g*, and *Kdm6b*) have also been linked to developmental delay and intellectual disabilities. Additionally, *Akap11* and *Kdm6b* have been implicated in bipolar disorder and autism spectrum disorder, respectively [5, 48, 49].

To determine whether DEGs from the CNV models are enriched for schizophrenia SNP-heritability, we performed stratified linkage disequilibrium score (S-LDSC) analysis on mouse homologs. Among the cell type-specific DEGs with sufficient SNPs for robust estimation, L6 corticothalamic projection neurons showed enrichment for schizophrenia-associated SNPs in the 15q13.3del and 16p11.2dup mouse models (Fig. 2B). We also tested for enrichment across neurodevelopmental and cognitive traits associated with these CNV syndromes, including attention deficit/hyperactivity disorder, autism spectrum disorder, developmental delay, and intelligence (Supplementary Fig. 9B). In addition to schizophrenia, DEGs from L6 corticothalamic projection neurons in the 15q13.3del and 16p11.2dup models were also enriched for intelligence-associated SNPs, alongside L4/5 and L6 intratelencephalic projection neurons from 3q29del and 15q13.3del models, respectively.

DEGs from 16p11.2dup L6 corticothalamic neurons were enriched for neuron differentiation, generation of precursor metabolites and energy, and regulation of programmed cell death (Supplementary Fig. 9C). DEGs from 15q13.3del L6 corticothalamic neurons were enriched for synaptic signaling and transmission, synapse organization, and neurogenesis (Supplementary Fig. 9D). Further investigation of these DEGs revealed an enrichment of genes associated with some of the phenotypic abnormalities reported in 15q13.3 deletion syndrome based on Human Phenotype Ontology terms, including seizures, irregular electroencephalogram abnormalities, and abnormal behaviors such as repetitive movements and aggression (Fig. 2C). This suggests that the same cell type contributes to both schizophrenia risk and the neurological and behavioral abnormalities seen in patients with 15q13.3 deletion syndrome, potentially offering a promising avenue for understanding its underlying biological mechanisms.

### Analysis of convergence of DEGs across models

Next, we investigated the extent to which DEGs are shared across the different CNV models. Cell type-specific analysis revealed remarkably little overlap among the models: overall, 97% of all identified DEGs were not shared across any mouse models, highlighting the distinctiveness of the perturbations associated with each CNV (Supplementary Fig. 10A-D***)***. However, an exception to this trend was observed in L6 corticothalamic projection neurons, where 124 DEGs were shared between 15q13.3del and 3q29del (significantly more than expected by chance, *p* < 0.001, hypergeometric test), indicating some convergence, albeit between only two of five models (Fig. 2D). These shared DEGs were associated with synaptic signaling and neuron development processes (Fig. 2E). Additionally, at a pseudobulk level (merged cell types), no dysregulated genes were shared among four or five CNV models (Supplementary Fig. 10E-F).

**Fig. 3:**
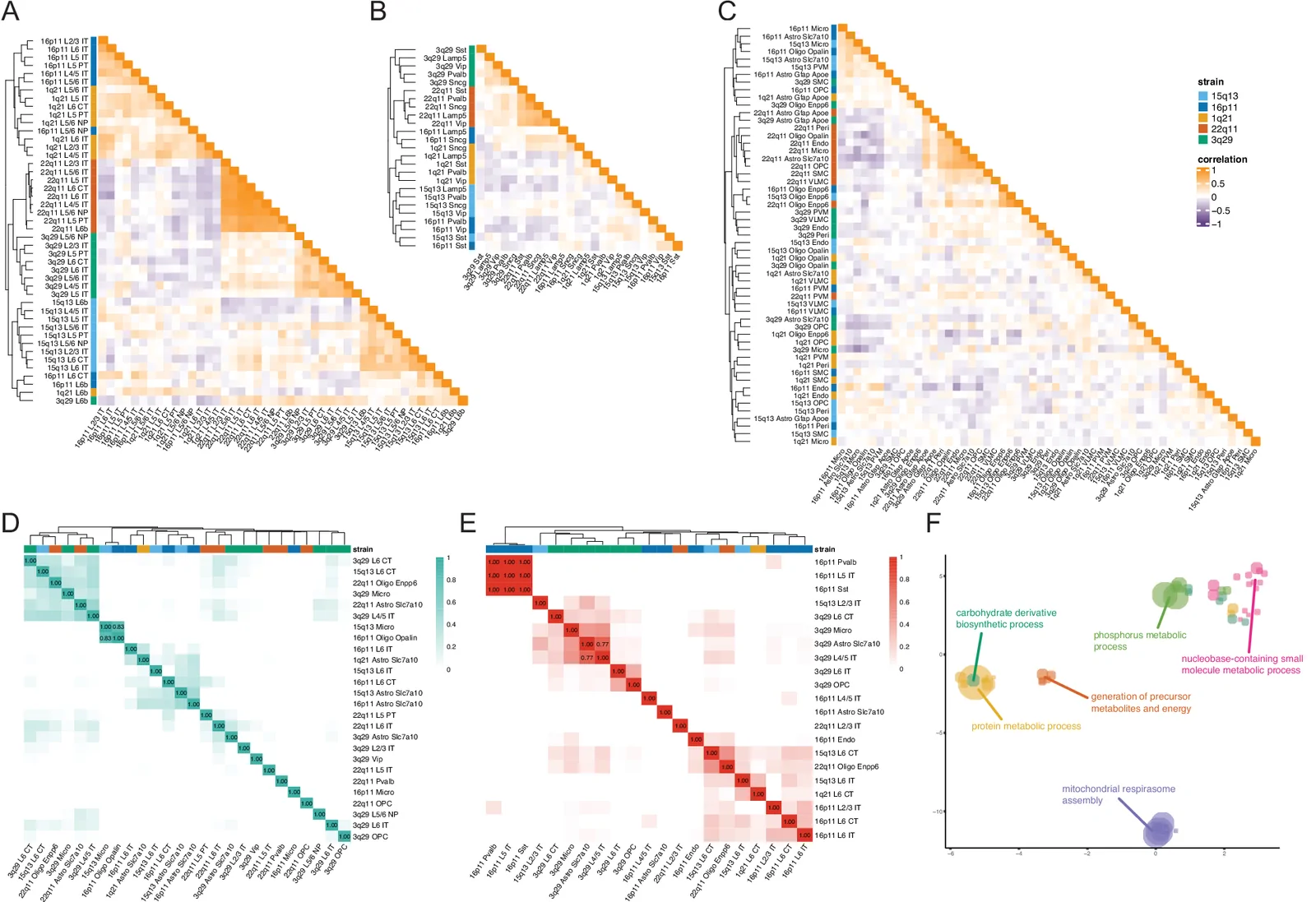
CNV models drive unique patterns of transcriptional dysregulation. **A** Glutamatergic neurons, (**B**) GABAergic neurons, and (**C**) non-neuronal cells. For each cell class, pairwise Pearson correlations were computed between all CNV model and cell type comparisons, based on mean log2 fold changes of the top 100 most differentially expressed genes per cell type (lowest adjusted p-values), followed by hierarchical clustering to assess expression concordance across conditions. **D, E** Hierarchically clustered heatmap of semantic similarity values calculated between pairs of GO terms overrepresented in downregulated (**D**) and upregulated (**E**) DEGs. **F** GO terms shared between astrocytes and L4-5 IT neurons from 3q29 mice, reduced based on their semantic similarity. Distances between points represent the similarity between terms and point sizes represent the number of genes contained in each GO term.

To assess consistency in gene dysregulation across CNV models, we selected the top 100 genes per cell type (ranked by lowest adjusted p-values) and computed pairwise Pearson correlations of their mean log2 fold changes. Correlation matrices were hierarchically clustered to reveal patterns across conditions. In glutamatergic neurons, this clustering almost entirely distinguishes the five CNV mouse models, indicating that transcriptomic changes are more similar within the same model than among neurons of the same subtype across different models (Fig. 3A). The clustering also revealed two main groups: 16p11.2dup clustered together with 1q21.1del, while 22q11.2del clustered with 3q29del, suggesting greater similarity within each pair. Notably, these two groups appear to be moderately anti-correlated, indicating opposing patterns of gene expression changes in glutamatergic neurons across CNV models. 15q13.3del formed a distinct branch, suggesting a unique expression profile relative to the other models. A similar pattern of clustering by CNV model was observed in GABAergic neurons (Fig. 3B). In contrast, non-neuronal cells did not cluster by CNV model or cell type, apart from the 22q11.2del model, which was mostly distinct from the other models (Fig. 3C). Interestingly, differentiation-committed oligodendrocyte precursors (Oligo Enpp6) from 16p11.2dup, 15q13.3del, and 22q11.2del clustered together and were negatively correlated with those from 3q29del and 1q21.1del. These opposing patterns of gene expression suggest that 16p11.2dup, 15q13.3del, and 22q11.2del models may share similar molecular changes in this cell type that contrast with those in 3q29del and 1q21.1del models, potentially reflecting distinct mechanisms of cellular dysfunction.

### Convergence on the level of biological pathways

Although the five CNV models appear to exhibit unique patterns of gene expression, it is possible that gene expression changes in each CNV model coalesce onto the same biological processes. To investigate this, we performed GO term enrichment analysis on up- and down-regulated genes from each model and cell type, and then used a semantic similarity approach to assess relatedness between GO term sets [50]. Remarkably, most enriched terms were specific to their respective cell types and CNV models, with few or no shared biological processes across groups (Fig. 3D, E). For example, in the 16p11.2dup model, processes relating to blood vessel development and morphogenesis were upregulated in endothelial cells, a phenotype not observed in other models (Supplementary Fig. 7E). This finding is particularly notable given that endothelial dysfunction resulting in impaired cerebral angiogenesis has been previously reported in mice harboring the 16p11.2 deletion [51].

While such distinct patterns dominated, one notable exception was observed: microglia from the 15q13.3del model and oligodendrocytes from the 16p11.2dup model both showed downregulation of terms related to cytoplasmic translation. In both cases, this enrichment was driven by a significant over-representation of downregulated ribosomal genes, suggesting that protein synthesis is suppressed in these cells. Regarding upregulated terms, the highest similarity was observed among cell types from the 16p11.2dup model: Pvalb interneurons, Sst interneurons, and L5 intratelencephalic neurons. This similarity was driven by the shared enrichment of telomere maintenance processes, likely driven by the involvement of several genes within the 16p11.2 locus. A high degree of similarity was also discovered between astrocytes and L4/5 intratelencephalic neurons from the 3q29del model. This similarity was driven by activation of metabolic processes, including ATP and nucleotide synthesis, translation, respiratory chain complex assembly, and energy generation (Fig. 3F), consistent with previous reports of gene dysregulation in mitochondria as a consequence of the 3q29 deletion [52].

### Transcriptional alterations are developmentally dynamic

**Fig. 4:**
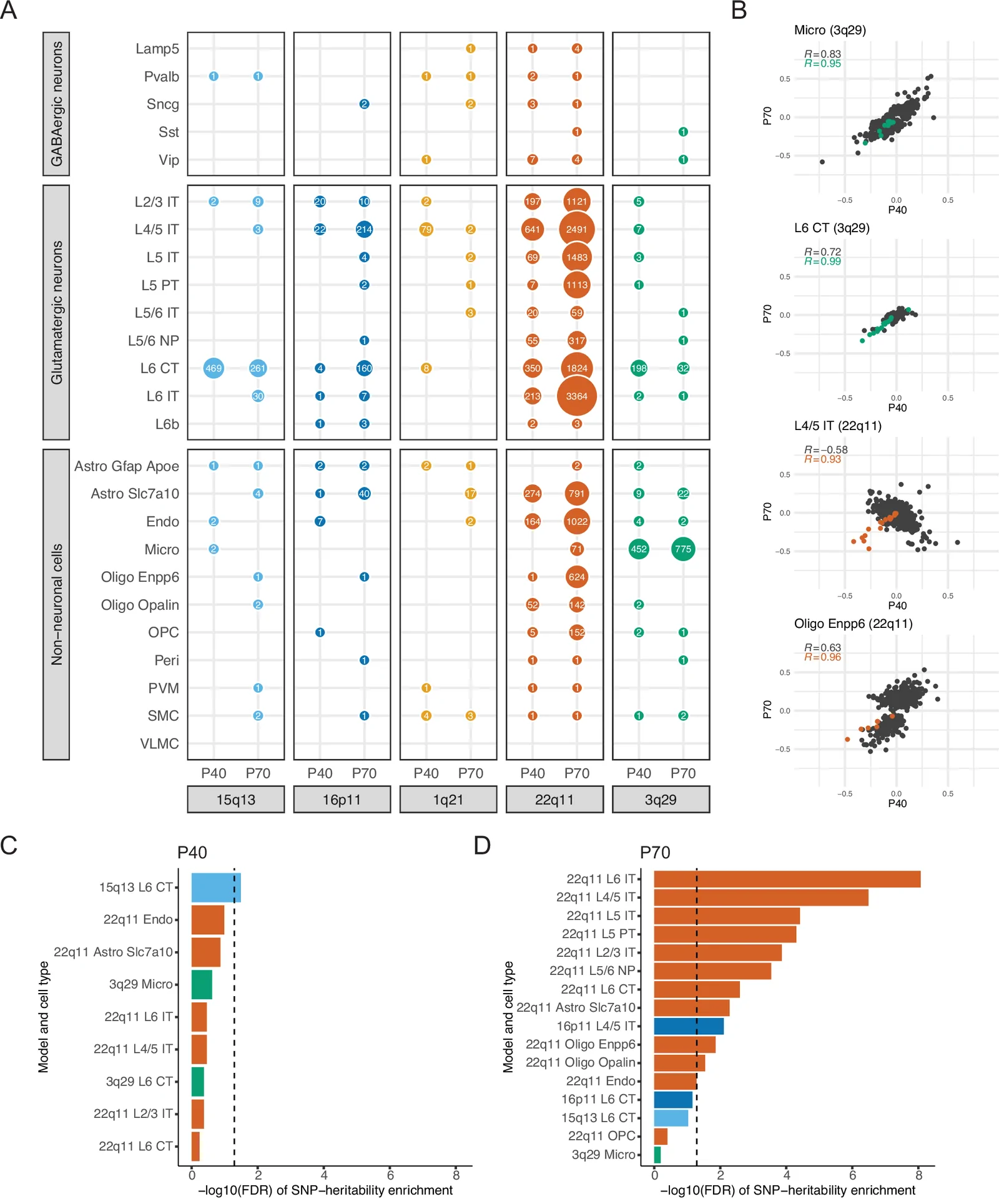
Each CNV model displays unique temporal dynamics, with divergent age effects in 22q11.2del mice associated with schizophrenia genetic risk in adulthood. **A**. Total number of significant DEGs (FDR < 0.1), excluding genes in the CNV regions, identified in each age group and cell type when comparing mutant mice to wildtype mice. **B** Example comparisons of mean log2 fold change values for DEGs between age groups. Pearson correlation coefficients are calculated separately for CNV locus genes (colored) and other DEGs (black). **C, D** Significance of SNP-heritability enrichment in DEG lists from P40 (**C**) and P70 (**D**) mice. Only DEG lists that contain sufficient numbers of SNPs ( ≥ 1%) to yield robust estimates are shown.

Since schizophrenia typically emerges during the transition into early adulthood, we next asked whether transcriptional dysregulation associated with each CNV is already present at late adolescence or arises during this critical developmental window. Analyzing the adolescent and adult mice separately revealed both age- and cell-type-specific effects (Supplementary Data File 1). In most models, the number of DEGs increased in early adulthood, suggesting an intensification of transcriptional dysregulation with age (Fig. 4A). While in the joint analysis differentiation-committed oligodendrocyte precursors (Oligo Enpp6) were indicated as the most affected cell type in the 22q11.2del model, there was a much higher signal in excitatory neurons and other non-neuronal cells in the age-specific analysis. To investigate this discrepancy, we examined how the mean log2 fold changes of DEGs changed with age in perturbed cell types. Interestingly, the 22q11.2del model showed opposing patterns of dysregulation across ages, with predominantly negative correlations, except in the differentiation-committed oligodendrocyte precursors (Oligo Enpp6) (Fig. 4B*,* Supplementary Fig. 11). This explains why these cells emerged as most affected in the joint analysis and highlights substantial transcriptomic remodeling between adolescence and adulthood that can be obscured when analyzing the data across developmental time points.

This remodeling appears biologically meaningful: schizophrenia heritability enrichment analysis revealed that dysregulated genes in early adult but not adolescent 22q11.2del mice were enriched for schizophrenia risk (Fig. 4C, D). A similar, though more modest, trend was observed in the 16p11.2dup model, although these results may partly reflect differences in gene list size. Notably, while only one cell type in adolescent mice (L6 corticothalamic projection neurons from 15q13.3del) showed enrichment for ADHD heritability, we observed broader enrichment for multiple traits in the adult brain (Supplementary Fig. 12A-B). In adult 22q11.2del mice, DEGs from multiple excitatory neuron types and astrocytes were enriched for ADHD-, ASD-, and intelligence-associated SNPs. These findings suggest that 22q11.2 deletion-related transcriptional changes become more aligned with common variant risk architecture later in development.

**Fig. 5:**
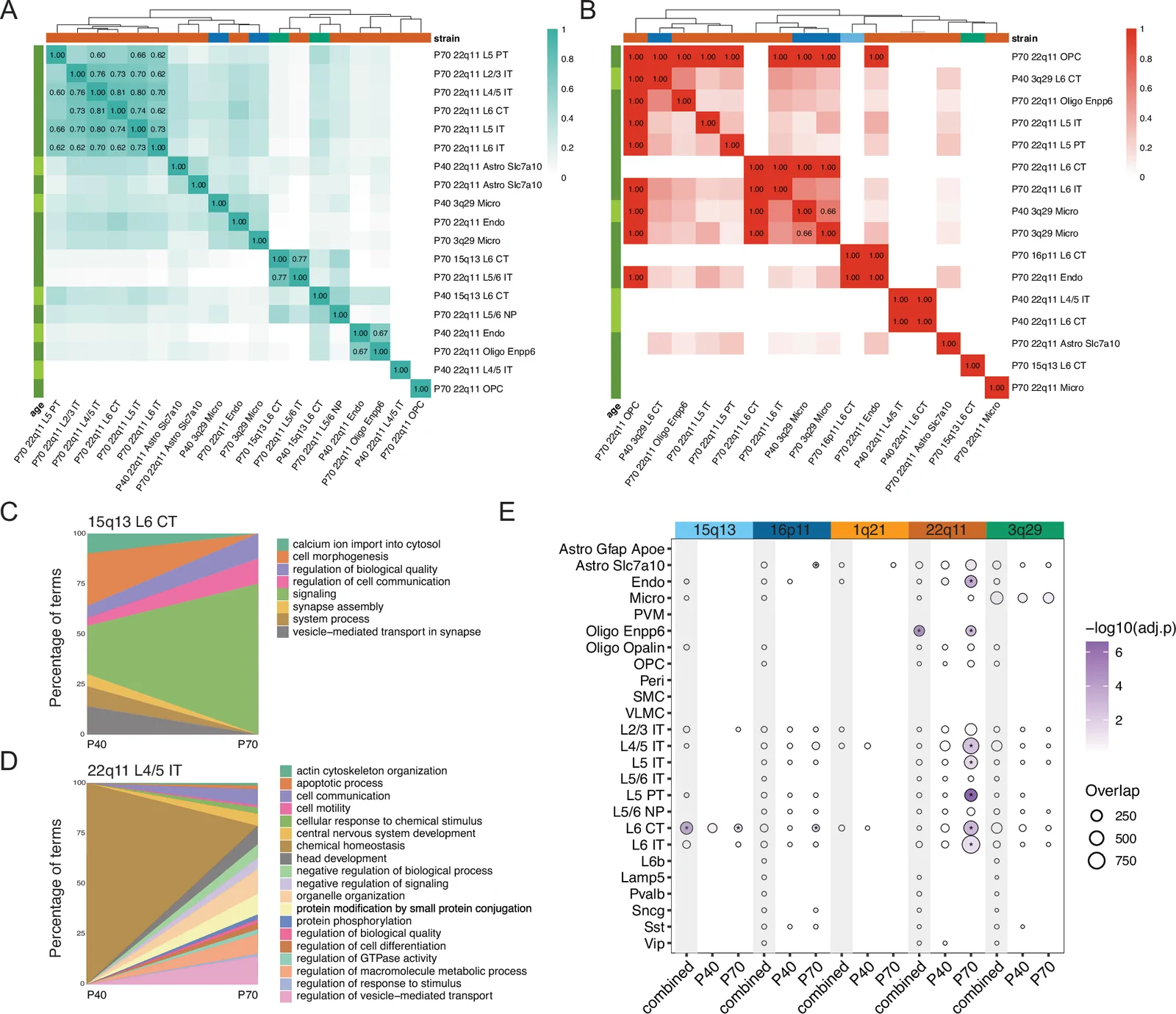
Despite limited cross-model overlap, transcriptional dysregulation in CNV models significantly overlaps with postmortem gene expression changes in schizophrenia. **A, B** Hierarchically clustered heatmap of semantic similarity values calculated between pairs of GO terms overrepresented in downregulated (**A**) and upregulated (**B**) DEGs. **C, D** GO terms over-represented in downregulated DEGs from L6 CT neurons from 15q13.3del mice (**C**) and L4-5 IT neurons from 22q11.2del mice (**D**). For readability, GO terms were reduced based on their semantic similarity and the percentage of terms from each age are plotted. **E** Significance of overlap between cell type-specific DEG sets identified in each CNV model and DEGs identified by bulk RNA-seq of postmortem cortical brain tissue from individuals with schizophrenia [54], computed with the SuperExactTest package [86]. Asterisks denote statistically significant overlaps after multiple comparison correction (p.adj < 0.05). Missing dots indicate not tested.

Across all models, gene dysregulation varied by cell type and age, with glutamatergic neurons showing the strongest model-specific effects (Supplementary Fig. 13A–F). Biological processes implicated by dysregulated genes were also largely model-specific, though some shared pathways emerged (Fig. 5A, B). For instance, synaptic signaling was downregulated in L6 CT and L5/6 IT excitatory neurons from 15q13.3del and 22q11.2del adult mice, respectively. Additionally, genes involved in more general biological processes such as translation and RNA processing were upregulated by OPCs and L6 CT neurons, respectively, from adult 22q11.2del mice, which were shared by several cell types from 3q29del mice. Within the 22q11.2del model, glutamatergic neurons from adult mice showed considerable overlap in downregulated gene ontology terms related to synaptic organization and cell communication. Complementary SynGO analysis [53] further implicated synaptic signaling, synapse assembly regulation, and postsynaptic density structure (Supplementary Fig. 13G), suggesting broad impairment of excitatory neuronal communication in the prefrontal cortex of 22q11.2del mice in young adulthood.

To better understand how transcriptional programs evolve over adolescent development, we focused on schizophrenia-relevant DEGs (from S-LDSC) and their enriched GO terms. In L6 corticothalamic projection neurons from 15q13.3del mice, adolescence was marked by widespread downregulation of processes like calcium ion import, neuron projection development and morphogenesis, and vesicle-mediated transport, suggesting early disruption of key developmental programs (Fig. 5C). In contrast, young adulthood produced fewer, more focused disruptions, mostly in signaling pathways, possibly reflecting a residual impairment after earlier dysregulation. Conversely, L4-5 intratelencephalic neurons from 22q11.2del mice showed biologically focused changes in adolescence followed by widespread downregulation in adulthood, including cell communication, cell motility, and vesicle-mediated transport (Fig. 5D). These findings indicate that the transition into adulthood is a particularly vulnerable developmental window in the context of 22q11.2 deletion, during which the function of glutamatergic neurons is disrupted in a schizophrenia-relevant manner.

Finally, we compared our DEG sets with postmortem brain studies in schizophrenia [54, 55] and iPSC-derived cortical organoids from individuals with CNV syndromes [56]. Despite little overlap between DEGs across CNV mouse models, most models showed significant overlap with dysregulated genes from both bulk (Fig. 5E) and single-nuclei RNA-seq data (Supplementary Fig. 14) from postmortem brain tissue from individuals with schizophrenia, across several distinct cell types. This overlap suggests that transcriptomic changes driven by distinct schizophrenia risk variants partially recapitulate the gene expression changes observed in the human transcriptomic phenotype. Fewer overlaps were observed with CNV cortical organoids (Supplementary Fig. 15), which may reflect differences in developmental maturity between organoids and the adolescent and adult mouse cortex used here.

## Discussion

Our study presents a single-cell transcriptomic comparison across five schizophrenia-associated CNV mouse models. We focused on the prefrontal cortex during the late adolescent and early adult period, when the disorder manifests in humans, and identified transcriptomic alterations specific to cell types and ages. Given the paucity of available single-cell datasets for most of these models, we also hope this will serve as a valuable resource for future CNV research.

We began with the hypothesis that schizophrenia-associated CNVs might converge on shared gene expression changes or biological processes within key cell types, and remained alert to the possibility that common DEGs might also be implicated by human genomic studies [6]. Contrary to our expectations, we found minimal overlap in DEGs or dysregulated pathways across CNV models. Given the accumulating evidence that rare loss-of-function deletions linked to autism spectrum disorder converge on common molecular pathways [57–61], our results challenge the notion of a similar pattern in schizophrenia-associated CNVs. These findings instead suggest that rare variants linked to schizophrenia act through distinct, CNV-specific mechanisms at the transcriptomic level.

Despite this molecular divergence, some cell types were more consistently affected. Excitatory neurons were significantly perturbed in several models and emerged as a key cell class of interest, consistent with prior studies showing that schizophrenia-associated common variants predominantly map to excitatory neurons in both humans and mouse models [8, 62, 63]. Layer 6 corticothalamic projection neurons were the most affected population in 1q21.1del, 16p11.2dup, and 15q13.3del mice, and the affected gene sets showed schizophrenia heritability enrichment in the latter two models and in adult 22q11.2del mice. These findings build on emerging data from postmortem human brain tissue implicating deep-layer excitatory neurons in schizophrenia [54, 64], reinforcing their potential role as a point of convergence across genetic variants. However, while certain deep-layer neuron types may be selectively vulnerable, the molecular routes to dysfunction seem to vary by genetic insult.

The timing of transcriptomic dysregulation also varied by CNV. In two models (16p11.2dup and 22q11.2del), dysregulation intensified with age, whereas the other three showed a more mixed pattern. Across most models, transcriptomic changes were directionally correlated between adolescence and adulthood, suggesting that disruptions persist over time. The 22q11.2del model was a notable exception, displaying anti-correlated gene expression changes across ages that reflect a striking shift in transcriptomic profile between adolescence and early adulthood. This molecular transition parallels previously reported developmental reconfigurations in synaptic architecture and functional connectivity, in which hyperconnectivity transitions to hypoconnectivity across puberty in both another 22q11.2del mouse model and human 22q11.2 deletion carriers [65]. The genes dysregulated in young adulthood were highly enriched for schizophrenia heritability and converged on synaptic signaling processes, consistent with prior findings implicating GSK3β-dependent synaptic alterations as a driver of connectivity changes during this period [65]. Together this suggests that the previously observed dysconnectivity may emerge from a coordinated developmental shift in synaptic gene expression and that this specific developmental window may be especially relevant for schizophrenia risk.

Overall, our findings demonstrate that the onset and progression of molecular dysregulation are CNV-specific, supporting a model in which ‘many roads lead to Rome’: rare CNVs all confer risk for schizophrenia via distinct molecular mechanisms. Yet, significant overlap between DEGs from CNV mice and postmortem brain tissue from individuals with schizophrenia indicates that these distinct transcriptomic changes are partially shared with broader gene expression dysregulation observed in humans, suggesting that different molecular paths may ultimately contribute to a shared phenotypic outcome.

While CNV-specific transcriptomic changes partially recapitulate gene expression changes in schizophrenia cases, convergence between CNV models themselves may occur at a different biological level, such as circuit-level dysfunction. Alternatively, CNVs might create a generalized vulnerability upon which additional risk factors, such as other genetic variants or environmental influences, act to promote convergence. Supporting this, individuals with schizophrenia who carry schizophrenia-associated CNVs have a higher burden of common risk alleles compared to non-affected CNV carriers [66], and experimental stress during young adulthood in the 15q13.3del mouse model exacerbates the behavioral phenotype [67]. The significant perturbation of deep layer excitatory neurons observed across CNV models may be particularly relevant here, as excitatory neurons are well-established targets of chronic stress paradigms [68].

Our study has several limitations. We focused on the prefrontal cortex, though future studies are certain to evaluate additional brain regions relevant to schizophrenia (e.g., other cortical areas, hippocampus, thalamus, and amygdala) [63, 69, 70]. Additionally, the CNV mouse models are maintained on inbred genetic backgrounds that lack the polygenic common variants present in humans, meaning that their contribution to disease mechanisms is not captured. While the mouse lacks a true dorsolateral PFC homologue, the prefrontal cortex-like regions present in mice share substantial molecular and connectivity features with their human counterparts [71]. Our findings, supported by the overlap with human transcriptomic studies, capture schizophrenia-relevant biology, though it remains possible that additional convergent effects might be human specific and could not be detected in our model system. Transcriptomic analysis of post-mortem brain tissues from individuals with CNVs would ideally address the latter limitations, though obtaining sufficient sample sizes for meaningful statistical inference will be challenging for the rarest CNVs.

In summary, we find that schizophrenia-associated CNVs do not converge at the molecular level in a baseline state, but instead exert distinct age- and cell type-specific disease-related transcriptomic effects. Our findings highlight the importance of developmental timing, support a potential role for deep-layer excitatory neurons, and emphasize the value of animal models for capturing cell vulnerability during adolescent development. Whether convergence emerges under stress or at other biological levels remains an open and important question.

## Methods

### Animals

All animal experimental procedures followed the guidelines and recommendations of Swedish animal protection legislation and were approved by the local ethical committee for experiments on laboratory animals. The 15q13.3del (model no. 10962) and 1q21.1del (model no. 11025) models were obtained from Taconic Biosciences. Both the 16p11.2 (EM:06134) and 22q11.2 (EM:02122) models were obtained through The European Mutant Mouse Archive (EMMA), provided by Thomas Arbogast and Antonio Baldini, respectively. The 3q29 model was generated and provided to our lab for inclusion in this project by Jennifer Mulle. All mouse models were maintained on a C57BL/6 N background. Experiments were conducted on male and female mice aged 40 and 70 days. A total of 80 mice were included in the study: for each CNV model, 8 mutants (4 per age group) and 8 littermate controls (4 per age group) were used.

### Single-cell dissociation

Mice harboring the CNV and control littermates were sacrificed with an overdose of ketamine/xylazine, followed by transcardial perfusion with artificial cerebrospinal fluid (aCSF; 93 mM NMDG, 2.5 mM KCl, 1.2 mM NaH_2_PO_4_, 30 mM NaHCO_3_, 20 mM HEPES, 25 mM glucose, 5 mM sodium ascorbate, 2 mM thiourea, 3 mM sodium pyruvate, 10 mM N-acetyl-L-cysteine, 10 mM MgSO_4_, 0.5 mM CaCl_2_; adjusted to pH 7.4). The aCSF was cooled at −80 °C for 40 min and equilibrated in 95% O_2_/5% CO_2_ before transcardial perfusion was performed. Brains were removed and 300 μm vibratome sections were collected. Dissected prefrontal cortex fragments, encompassing the secondary motor area, anterior cingulate area, prelimbic area, and infralimbic area, from one male and one female littermate were pooled together for subsequent steps. The prefrontal cortex fragments were dissociated using the Papain Dissociation System (Worthington) for 35 min at 37 °C. Following papain dissociation, the fragments were manually triturated using fire-polished Pasteur pipettes and centrifugation in albumin inhibitor. The cells were resuspended in cold aCSF solution (4 °C) with reduced MgSO_4_ (2.5 mM) and 1% bovine serum albumin and were then passed through a 20 μM filter (Miltenyi Biotec). Negative DRAQ7 (Abcam) events were FAC sorted on a BD FACSDiva 8.0.2 or BD FACSAria III and collected in 4 °C aCSF with reduced MgSO_4_ (2.5 mM) and 1% bovine serum albumin.

### Single-cell RNA-sequencing

Cell suspensions were visually inspected for viability. Library preparation was carried out using the Chromium Next GEM Single Cell Kit v3.1 (10x Genomics). Cell stock concentrations were selected based on a targeted recovery of 5000 cells per mouse. For downstream cDNA synthesis and library construction, the manufacturer’s instructions were followed. Libraries were sequenced by National Genomics Infrastructure (NGI) Sweden on Illumina NovaSeq6000 S4-200 v1.5 flow cells.

### Data pre-processing

The Illumina reads were demultiplexed and aligned to the mouse genome (refdata-gex-mm10-2020-A) using CellRanger v5.0.1 (10X Genomics). Cell-free mRNA contamination was removed using the SoupX package [72]. General pre-processing and clustering were performed using the Scanpy package [73]. Genes that were detected in fewer than 20 cells were excluded, retaining 25,478 for downstream analysis. Standard quality control criteria were applied to remove low-quality cells, including those with fewer than 500 genes, fewer than 1000 UMI counts, a mitochondrial gene fraction exceeding 10%, or predicted doublets identified using the Scrublet package [74]. Cell sex was predicted from *Xist* expression and cells that could not be confidently assigned were excluded. Normalization and cell type clustering were performed using the Scanpy package. UMI counts were normalized to 1,000,000 molecules per cell and log-transformed, followed by selection of the top 3000 highly variable genes. A nearest-neighbor graph was computed on the first 30 principal components, clustered using the Leiden algorithm, and projected to two dimensions using UMAP. Cell types were annotated using the scmapCell2Cluster function from the scmap package [75] using the Mouse Whole Cortex and Hippocampus 10X dataset from the Allen Institute for Brain Science as a reference (restricted to ACA, MOs;FRP, and PL;ILA;ORB regions), and validated against known marker genes. Clusters with ambiguous gene expression patterns, contributions from fewer than 80% of samples, or disproportionate contribution from a single sample were removed to ensure robust cell type annotations and to retain only well-represented cell types for downstream differential gene expression analysis.

### Cell composition analysis

To test for statistically significant differences in cell type proportions between wildtype and mutant mice from each mouse model, we used the propeller function from the speckle package in R [45]. Briefly, this function calculates cell type proportions for each sample, performs a variance stabilizing transformation on the proportions, and then tests for significant differences in transformed proportions between conditions using linear models. Multiple testing is corrected using Benjamini-Hochberg adjustment. We used the default settings and arcsin square root transformation of proportions.

### Differential gene expression analysis

An additional round of gene filtering was implemented prior to analysis to exclude genes that were expressed in fewer than 10% of cells per cell type. We then applied a negative binomial generalized linear mixed model, modified from the Libra package [46, 47]. The library size factors were incorporated as an offset term, the sample effect (individual mouse) was treated as a random factor, and the batch (mouse pair) and genotype were treated as fixed factors in the full model. The reduced model excluded the genotype fixed factor. The batch information was not included as a fixed factor in the analysis of the 22q11 mouse model since the pair information variable can be formed by the combination of other factor levels (Supplementary Note 1). The likelihood-ratio test was used to test the difference between the two models (full and reduced) using the Chi-square distribution and adjusted p-values were calculated using Benjamini-Hochberg adjustment.

### Pearson correlation analysis

To assess consistency in gene dysregulation across models, we selected the top 100 genes per cell type and model (ranked by lowest adjusted p-values) and computed pairwise Pearson correlations of their mean log2 fold changes. The resulting correlation matrices were transformed into dissimilarity matrices and used for unsupervised hierarchical clustering, visualized using the ComplexHeatmap package [76].

### Gene ontology enrichment analysis

Functional enrichment of differentially expressed genes was performed using gprofiler2 (version 0.2.2) [77]. Only queries with at least 5 genes were considered. The background set was limited to annotated genes expressed in the full dataset after filtering and the default method (gSCS) was used for multiple testing correction. GO terms were considered significantly enriched if the corrected p-value was below 0.05. GO term sets with more than 5000 members or less than 5 members were removed for the sake of interpretation. To calculate semantic similarity between sets of GO terms, we used the Wang method with the best-match average strategy, as implemented in the GOSemSim R package [50, 78]. The rrvgo package [79] was used to reduce and plot lists of GO terms using the default settings (similarity threshold = 0.7).

### Gene set analysis (GSA)

GSA was performed to identify significantly enriched human phenotype ontology (HPO) terms in gene lists obtained through DGE analysis (FDR < 0.1, only protein-coding genes) relative to a background gene set (genes present in the dataset). Hypergeometric tests were performed to assess the probability of observing the genes in each HPO term by chance. Multiple testing correction was applied using Bonferroni adjustments and results were considered significant if the corrected p-value was below 0.05.

### Heritability enrichment analysis

We evaluated the enrichment of common genetic variants associated with schizophrenia, ADHD, ASD, DD, and intelligence for the cell type-specific DEGs. For each cell type- and model-specific DEG list, we first identified 1-to-1 matched human homologs and mapped SNPs located within a 100 kb window around the start and end of each gene under the hg19/NCBI Build 37 coordinates. Next, for each gene list and trait, we performed enrichment analysis of the common genetic variants (GWAS summary statistics) [8, 80–83] processed with the munge.py function in the LDscore regression using stratified LDscore regression with the 1000 Genome European reference panel [84, 85]. We required the gene lists to contain ≥1% of the reference SNPs to consider the enrichment analysis result as robust. We derived p-values from the regression coefficients and applied FDR to correct for multiple testing for the robust DEG lists.

### Overlap analysis with published datasets

To assess overlap between DEGs from our dataset and published bulk and single-nuclei RNA-seq datasets [54–56], we used the SuperExactTest R package [86]. DEGs were defined using an adjusted p-value threshold of < 0.1. DEGs from all excitatory neuron subtypes in the published snRNA-seq dataset were pooled into a single gene set prior to testing to simplify the comparisons. Cell types with fewer than 10 DEGs were excluded. The background gene universe was defined as the union of all genes tested in both datasets. Multiple testing correction was performed using the Benjamini-Hochberg method. For bulk RNA-seq and snRNA-seq comparisons, correction was applied jointly across all CNV models and cell types within each age group. For iPSC bulk RNA-seq comparisons, correction was applied jointly across all CNV models and cell types within each age group and timepoint combination. Adjusted p-values < 0.05 were considered significant.

### Statistical analysis and study design

No statistical methods were used to predetermine sample size. Prior to library preparation, single cell suspensions were excluded if the cell viability was below 50%. After sequencing, cells were excluded from the analysis following the filtering steps outlined under data pre-processing. No animals were excluded from the study. Animals were randomized within genotype groups using their identification numbers. Mutant and wildtype littermates were processed concurrently within the same paired experimental batches. Because mice from both genotypes were pooled and handled together throughout library preparation and sequencing, differential treatment of mice based on genotype was not possible. Data pre-processing was applied uniformly across all mice using identical parameters regardless of strain or genotype identity. Statistical analyses were performed using established best-practice methods for single-cell RNA-sequencing, employing models appropriate for sparse, high-dimensional count data that do not assume normality or equal variance across groups. For each figure panel involving statistical comparison, the adjusted p-values are indicated in the figure legend and the methods used for multiple comparison correction are reported in the corresponding methods sections. Formal estimates of within-group variation were not calculated, as these metrics are not standard in single-cell transcriptomic analyses.

## Supplementary information

Supplementary Materials

Supplementary Data File 1

## Data Availability

The data discussed in this publication have been deposited in NCBI’s Gene Expression Omnibus and are publicly available through GEO Series accession number GSE329215.

## Code availability

Code used for data pre-processing and analysis is available at https://github.com/Hjerling-Leffler-Lab/SchizophreniaCNVAnalysis.

## References

[1] Jauhar S, Johnstone M, McKenna PJ. Schizophrenia. Lancet. 2022;399:473–86. 10.1016/S0140-6736(21)01730-X 35093231

[2] Kirkbride JB, Errazuriz A, Croudace TJ, Morgan C, Jackson D, Boydell J, et al. Incidence of schizophrenia and other psychoses in england, 1950–2009: a systematic review and meta-analyses. PLoS ONE. 2012;7:e31660 10.1371/journal.pone.0031660 PMC3310436 22457710

[3] Marshall CR, Howrigan DP, Merico D, Thiruvahindrapuram B, Wu W, Greer DS, et al. Contribution of copy number variants to schizophrenia from a genome-wide study of 41,321 subjects. Nat Genet. 2017;49:1 10.1038/ng.3725 PMC5737772 27869829

[4] Owen MJ, Legge SE, Rees E, Walters JTR, O’Donovan MC. Genomic findings in schizophrenia and their implications. Mol Psychiatry. 2023;28:3638–47. 10.1038/s41380-023-02293-8 PMC10730422 37853064

[5] Singh T, Poterba T, Curtis D, Akil H, Al Eissa M, Barchas JD, et al. Rare coding variants in ten genes confer substantial risk for schizophrenia. Nature. 2022;604:7906 10.1038/s41586-022-04556-w PMC9805802 35396579

[6] Sullivan PF, Yao S, Hjerling-Leffler J. Schizophrenia genomics: genetic complexity and functional insights. Nat Rev Neurosci. 2024;25:611–24. 10.1038/s41583-024-00837-7 39030273

[7] Sullivan PF, Geschwind DH. Defining the genetic, genomic, cellular, and diagnostic architectures of psychiatric disorders. Cell. 2019;177:162–83. 10.1016/j.cell.2019.01.015 PMC6432948 30901538

[8] Trubetskoy V, Pardiñas AF, Qi T, Panagiotaropoulou G, Awasthi S, Bigdeli TB, et al. Mapping genomic loci implicates genes and synaptic biology in schizophrenia. Nature. 2022;604:7906 10.1038/s41586-022-04434-5 PMC9392466 35396580

[9] Bassett AS, Chow EWC, AbdelMalik P, Gheorghiu M, Husted J, Weksberg R. The schizophrenia phenotype in 22q11 deletion syndrome. Am J Psychiatry. 2003;160:1580–6. 10.1176/appi.ajp.160.9.1580 PMC3276594 12944331

[10] Flaherty E, Zhu S, Barretto N, Cheng E, Deans MP, Fernando M, et al. Neuronal impact of patient-specific aberrant NRXN1α splicing. Nat Genet. 2019;51:1679–90. 10.1038/s41588-019-0539-z PMC7451045 31784728

[11] Khan TA, Revah O, Gordon A, Yoon SJ, Krawisz AK, Goold C, et al. Neuronal defects in a human cellular model of 22q11.2 deletion syndrome. Nat Med. 2020;26:1888–98. 10.1038/s41591-020-1043-9 PMC8525897 32989314

[12] Nehme R, Pietiläinen O, Artomov M, Tegtmeyer M, Valakh V, Lehtonen L, et al. The 22q11.2 region regulates presynaptic gene-products linked to schizophrenia. Nat Commun. 2022;13:3690 10.1038/s41467-022-31436-8 PMC9237031 35760976

[13] Pak C, Danko T, Mirabella VR, Wang J, Liu Y, Vangipuram M, et al. Cross-platform validation of neurotransmitter release impairments in schizophrenia patient-derived NRXN1-mutant neurons. Proc Natl Acad Sci USA. 2021;118:e2025598118 10.1073/pnas.2025598118 PMC8179243 34035170

[14] Parnell E, Culotta L, Forrest MP, Jalloul HA, Eckman BL, Loizzo DD, et al. Excitatory dysfunction drives network and calcium handling deficits in 16p11.2 duplication schizophrenia induced pluripotent stem cell–derived neurons. Biol Psychiatry. 2023;94:153–63. 10.1016/j.biopsych.2022.11.005 PMC10166768 36581494

[15] Arbogast T, Ouagazzal AM, Chevalier C, Kopanitsa M, Afinowi N, Migliavacca E, et al. Reciprocal effects on neurocognitive and metabolic phenotypes in mouse models of 16p11.2 deletion and duplication syndromes. PLOS Genet. 2016;12:e1005709 10.1371/journal.pgen.1005709 PMC4752317 26872257

[16] Baba M, Yokoyama K, Seiriki K, Naka Y, Matsumura K, Kondo M, et al. Psychiatric-disorder-related behavioral phenotypes and cortical hyperactivity in a mouse model of 3q29 deletion syndrome. Neuropsychopharmacology. 2019;44:2125–35. 10.1038/s41386-019-0441-5 PMC6887869 31216562

[17] Blizinsky KD, Diaz-Castro B, Forrest MP, Schürmann B, Bach AP, Martin-de-Saavedra MD, et al. Reversal of dendritic phenotypes in 16p11.2 microduplication mouse model neurons by pharmacological targeting of a network hub. Proc Natl Acad Sci. 2016;113:8520–5. 10.1073/pnas.1607014113 PMC4968749 27402753

[18] Blumenthal I, Ragavendran A, Erdin S, Klei L, Sugathan A, Guide JR, et al. Transcriptional consequences of 16p11.2 deletion and duplication in mouse cortex and multiplex autism families. Am J Hum Genet. 2014;94:870–83. 10.1016/j.ajhg.2014.05.004 PMC4121471 24906019

[19] Dachtler J, Ivorra JL, Rowland TE, Lever C, Rodgers RJ, Clapcote SJ. Heterozygous deletion of α-neurexin I or α-neurexin II results in behaviors relevant to autism and schizophrenia. Behav Neurosci. 2015;129:765–76. 10.1037/bne0000108 PMC4655861 26595880

[20] Didriksen M, Fejgin K, Nilsson SRO, Birknow MR, Grayton HM, Larsen PH, et al. Persistent gating deficit and increased sensitivity to NMDA receptor antagonism after puberty in a new mouse model of the human 22q11.2 microdeletion syndrome: a study in male mice. J Psychiatry Neurosci. 2017;42:48–58. 10.1503/jpn.150381 PMC5373712 27391101

[21] Drew LJ, Crabtree GW, Markx S, Stark KL, Chaverneff F, Xu B, et al. The 22q11.2 microdeletion: Fifteen years of insights into the genetic and neural complexity of psychiatric disorders. Int J Dev Neurosci. 2011;29:259–81. 10.1016/j.ijdevneu.2010.09.007 PMC3074020 20920576

[22] Etherton MR, Blaiss CA, Powell CM, Südhof TC. Mouse neurexin-1α deletion causes correlated electrophysiological and behavioral changes consistent with cognitive impairments. Proc Natl Acad Sci. 2009;106:17998–8003. 10.1073/pnas.0910297106 PMC2764944 19822762

[23] Fejgin K, Nielsen J, Birknow MR, Bastlund JF, Nielsen V, Lauridsen JB, et al. A mouse model that recapitulates cardinal features of the 15q13.3 microdeletion syndrome including schizophrenia- and epilepsy-related alterations. Biol Psychiatry. 2014;76:128–37. 10.1016/j.biopsych.2013.08.014 24090792

[24] Forsingdal A, Fejgin K, Nielsen V, Werge T, Nielsen J. 15q13.3 homozygous knockout mouse model display epilepsy-, autism- and schizophrenia-related phenotypes. Transl Psychiatry. 2016;6:e860. 10.1038/tp.2016.125 PMC5545711 27459725

[25] Gass N, Weber-Fahr W, Sartorius A, Becker R, Didriksen M, Stensbøl TB, et al. An acetylcholine alpha7 positive allosteric modulator rescues a schizophrenia-associated brain endophenotype in the 15q13.3 microdeletion, encompassing *CHRNA*7. Eur Neuropsychopharmacol. 2016;26:1150–60. 10.1016/j.euroneuro.2016.03.013 27061851

[26] Grayton HM, Missler M, Collier DA, Fernandes C. Altered social behaviours in neurexin 1α knockout mice resemble core symptoms in neurodevelopmental disorders. PLOS ONE. 2013;8:e67114 10.1371/journal.pone.0067114 PMC3696036 23840597

[27] Horev G, Ellegood J, Lerch JP, Son YEE, Muthuswamy L, Vogel H, et al. Dosage-dependent phenotypes in models of 16p11.2 lesions found in autism. Proc Natl Acad Sci. 2011;108:17076–81. 10.1073/pnas.1114042108 PMC3193230 21969575

[28] Kogan JH, Gross AK, Featherstone RE, Shin R, Chen Q, Heusner CL, et al. Mouse model of chromosome 15q13.3 microdeletion syndrome demonstrates features related to autism spectrum disorder. J Neurosci. 2015;35:16282–94. 10.1523/JNEUROSCI.3967-14.2015 PMC6605504 26658876

[29] Long JM, LaPorte P, Merscher S, Funke B, Saint-Jore B, Puech A, et al. Behavior of mice with mutations in the conserved region deleted in velocardiofacial/DiGeorge syndrome. Neurogenetics. 2006;7:247–57. 10.1007/s10048-006-0054-0 16900388

[30] Meechan DW, Maynard TM, Tucker ES, Fernandez A, Karpinski BA, Rothblat LA, et al. Modeling a model: mouse genetics, 22q11.2 deletion syndrome, and disorders of cortical circuit development. Prog Neurobiol. 2015;130:1–28. 10.1016/j.pneurobio.2015.03.004 PMC5019355 25866365

[31] Merscher S, Funke B, Epstein JA, Heyer J, Puech A, Lu MM, et al. *TBX1* is responsible for cardiovascular defects in velo-cardio-facial/digeorge syndrome. Cell. 2001;104:619–29. 10.1016/S0092-8674(01)00247-1 11239417

[32] Nielsen J, Fejgin K, Sotty F, Nielsen V, Mørk A, Christoffersen CT, et al. A mouse model of the schizophrenia-associated 1q21.1 microdeletion syndrome exhibits altered mesolimbic dopamine transmission. Transl Psychiatry. 2017;7:1–12. 10.1038/s41398-017-0011-8 PMC5802512 29187755

[33] Nilsson SRO, Celada P, Fejgin K, Thelin J, Nielsen J, Santana N, et al. A mouse model of the 15q13.3 microdeletion syndrome shows prefrontal neurophysiological dysfunctions and attentional impairment. Psychopharmacology. 2016;233:2151–63. 10.1007/s00213-016-4265-2 PMC4869740 26983414

[34] Nilsson SRO, Fejgin K, Gastambide F, Vogt MA, Kent BA, Nielsen V, et al. Assessing the cognitive translational potential of a mouse model of the 22q11.2 microdeletion syndrome. Cereb Cortex. 2016;26:3991–4003. 10.1093/cercor/bhw229 PMC5028007 27507786

[35] Rutkowski TP, Purcell RH, Pollak RM, Grewenow SM, Gafford GM, Malone T, et al. Behavioral changes and growth deficits in a CRISPR engineered mouse model of the schizophrenia-associated 3q29 deletion. Mol Psychiatry. 2021;26:772–83. 10.1038/s41380-019-0413-5 PMC6788962 30976085

[36] Stark KL, Xu B, Bagchi A, Lai WS, Liu H, Hsu R, et al. Altered brain microRNA biogenesis contributes to phenotypic deficits in a 22q11-deletion mouse model. Nat Genet. 2008;40:751–60. 10.1038/ng.138 18469815

[37] Chawner SJ, Watson CJ, Owen MJ. Clinical evaluation of patients with a neuropsychiatric risk copy number variant. Curr Opin Genet Dev. 2021;68:26–34. 10.1016/j.gde.2020.12.012 PMC8219523 33461126

[38] Szecówka K, Misiak B, Łaczmańska I, Frydecka D, Moustafa AA. Copy number variations and schizophrenia. Mol Neurobiol. 2023;60:1854–64. 10.1007/s12035-022-03185-8 PMC9984510 36580197

[39] Siletti K, Hodge R, Mossi Albiach A, Lee KW, Ding SL, Hu L, et al. Transcriptomic diversity of cell types across the adult human brain. Science. 2023;382:eadd7046 10.1126/science.add7046 37824663

[40] Yao Z, van Velthoven CTJ, Kunst M, Zhang M, McMillen D, Lee C, et al. A high-resolution transcriptomic and spatial atlas of cell types in the whole mouse brain. Nature. 2023;624:317–32. 10.1038/s41586-023-06812-z PMC10719114 38092916

[41] Gordon A, Forsingdal A, Klewe IV, Nielsen J, Didriksen M, Werge T, et al. Transcriptomic networks implicate neuronal energetic abnormalities in three mouse models harboring autism and schizophrenia-associated mutations. Mol Psychiatry. 2021;26:5 10.1038/s41380-019-0576-0 31705054

[42] Arellano JI, Duque A, Rakic P. A coming-of-age story: adult neurogenesis or adolescent neurogenesis in rodents? Front Neurosci. 2024;18:1383728 10.3389/fnins.2024.1383728 PMC10948509 38505771

[43] Cerase A, Pintacuda G, Tattermusch A, Avner P. Xist localization and function: new insights from multiple levels. Genome Biol. 2015;16:166 10.1186/s13059-015-0733-y PMC4539689 26282267

[44] Zhang M, Pan X, Jung W, Halpern AR, Eichhorn SW, Lei Z, et al. Molecularly defined and spatially resolved cell atlas of the whole mouse brain. Nature. 2023;624:343–54. 10.1038/s41586-023-06808-9 PMC10719103 38092912

[45] Phipson B, Sim CB, Porrello ER, Hewitt AW, Powell J, Oshlack A. propeller: testing for differences in cell type proportions in single cell data. Bioinformatics. 2022;38:4720–6. 10.1093/bioinformatics/btac582 PMC9563678 36005887

[46] Crowell HL, Soneson C, Germain PL, Calini D, Collin L, Raposo C, et al. muscat detects subpopulation-specific state transitions from multi-sample multi-condition single-cell transcriptomics data. Nat Commun. 2020;11:6077 10.1038/s41467-020-19894-4 PMC7705760 33257685

[47] Squair JW, Gautier M, Kathe C, Anderson MA, James ND, Hutson TH, et al. Confronting false discoveries in single-cell differential expression. Nat Commun. 2021;12:5692 10.1038/s41467-021-25960-2 PMC8479118 34584091

[48] Palmer DS, Howrigan DP, Chapman SB, Adolfsson R, Bass N, Blackwood D, et al. Exome sequencing in bipolar disorder identifies AKAP11 as a risk gene shared with schizophrenia. Nat Genet. 2022;54:541–7. 10.1038/s41588-022-01034-x PMC9117467 35410376

[49] Satterstrom FK, Kosmicki JA, Wang J, Breen MS, De Rubeis S, An JY, et al. Large-Scale exome sequencing study implicates both developmental and functional changes in the neurobiology of autism. Cell. 2020;180:568–584.e23. 10.1016/j.cell.2019.12.036 PMC7250485 31981491

[50] Yu G, Li F, Qin Y, Bo X, Wu Y, Wang S. GOSemSim: an R package for measuring semantic similarity among GO terms and gene products. Bioinformatics. 2010;26:976–8. 10.1093/bioinformatics/btq064 20179076

[51] Ouellette J, Toussay X, Comin CH, Costa LdaF, Ho M, Lacalle-Aurioles M, et al. Vascular contributions to 16p11.2 deletion autism syndrome modeled in mice. Nat Neurosci. 2020;23:1090–101. 10.1038/s41593-020-0663-1 32661394

[52] Purcell RH, Sefik E, Werner E, King AT, Mosley TJ, Merritt-Garza ME, et al. Cross-species analysis identifies mitochondrial dysregulation as a functional consequence of the schizophrenia-associated 3q29 deletion. Sci Adv. 2023;9:eadh0558 10.1126/sciadv.adh0558 PMC10431714 37585521

[53] Koopmans, Nierop F, van P, Andres-Alonso M, Byrnes A, Cijsouw T, et al. SynGO: an evidence-based, expert-curated knowledge base for the synapse. Neuron. 2019;103:217–234.e4. 10.1016/j.neuron.2019.05.002 PMC6764089 31171447

[54] Ruzicka WB, Mohammadi S, Fullard JF, Davila-Velderrain J, Subburaju S, Tso DR, et al. Single-cell multi-cohort dissection of the schizophrenia transcriptome. Science. 2024;384:eadg5136 10.1126/science.adg5136 PMC12772489 38781388

[55] Gandal MJ, Zhang P, Hadjimichael E, Walker RL, Chen C, Liu S, et al. Transcriptome-wide isoform-level dysregulation in ASD, schizophrenia, and bipolar disorder. Science. 2018;362:eaat8127 10.1126/science.aat8127 PMC6443102 30545856

[56] Gordon A, Yoon SJ, Bicks LK, Martín JM, Pintacuda G, Arteaga S, et al. Developmental convergence and divergence in human stem cell models of autism. Nature. 2026;651:1–13. 10.1038/s41586-025-10047-5 PMC12999519 41611887

[57] Paulsen B, Velasco S, Kedaigle AJ, Pigoni M, Quadrato G, Deo AJ, et al. Autism genes converge on asynchronous development of shared neuron classes. Nature. 2022;602:268–73. 10.1038/s41586-021-04358-6 PMC8852827 35110736

[58] Liao C, Moyses-Oliveira M, De Esch CEF, Bhavsar R, Nuttle X, Li A, et al. Convergent coexpression of autism-associated genes suggests some novel risk genes may not be detectable in large-scale genetic studies. Cell Genomics. 2023;3:100277 10.1016/j.xgen.2023.100277 PMC10112287 37082147

[59] Weinschutz Mendes H, Neelakantan U, Liu Y, Fitzpatrick SE, Chen T, Wu W, et al. High-throughput functional analysis of autism genes in zebrafish identifies convergence in dopaminergic and neuroimmune pathways. Cell Rep. 2023;42:112243 10.1016/j.celrep.2023.112243 PMC10277173 36933215

[60] Lalli MA, Avey D, Dougherty JD, Milbrandt J, Mitra RD. High-throughput single-cell functional elucidation of neurodevelopmental disease-associated genes reveals convergent mechanisms altering neuronal differentiation. Genome Res. 2020;30:1317–31. 10.1101/gr.262295.120 PMC7545139 32887689

[61] Willsey HR, Exner CRT, Xu Y, Everitt A, Sun N, Wang B, et al. Parallel in vivo analysis of large-effect autism genes implicates cortical neurogenesis and estrogen in risk and resilience. Neuron. 2021;109:1409 10.1016/j.neuron.2021.03.030 PMC8145999 33887193

[62] Skene NG, Bryois J, Bakken TE, Breen G, Crowley JJ, Gaspar HA, et al. Genetic identification of brain cell types underlying schizophrenia. Nat Genet. 2018;50:6 10.1038/s41588-018-0129-5 PMC6477180 29785013

[63] Yao S, Harder A, Darki F, Chang YW, Li A, Nikouei K, et al. Connecting genomic results for psychiatric disorders to human brain cell types and regions reveals convergence with functional connectivity. Nat Commun. 2025;16:395 10.1038/s41467-024-55611-1 PMC11700164 39755698

[64] Bast L, Yao S, Martínez-López JA, Memic F, French H, Valiukonyte M, et al. Transcriptomic and genetic analysis suggests a role for mitochondrial dysregulation in schizophrenia. medRxiv; p. 2025.03.14.25323827 [Preprint]. 2025 [cited 2025 May 21]. Available from: https://www.medrxiv.org/content/10.1101/2025.03.14.25323827v1.

[65] Alvino FG, Gini S, Minetti A, Pagani M, Sastre-Yagüe D, Barsotti N, et al. Synaptic-dependent developmental dysconnectivity in 22q11.2 deletion syndrome. Sci Adv. 2025;11:eadq2807 10.1126/sciadv.adq2807 PMC11900866 40073125

[66] Tansey KE, Rees E, Linden DE, Ripke S, Chambert KD, Moran JL, et al. Common alleles contribute to schizophrenia in CNV carriers. Mol Psychiatry. 2016;21:8 10.1038/mp.2015.143 PMC7609344 26643540

[67] Giovanoli S, Werge TM, Mortensen PB, Didriksen M, Meyer U. Interactive effects between hemizygous 15q13.3 microdeletion and peripubertal stress on adult behavioral functions. Neuropsychopharmacology. 2018;44:703 10.1038/s41386-018-0189-3 PMC6372643 30188511

[68] Magariños AM, McEwen BS. Stress-induced atrophy of apical dendrites of hippocampal CA3c neurons: comparison of stressors. Neuroscience. 1995;69:83–8. 10.1016/0306-4522(95)00256-i 8637635

[69] Aguilar DD, McNally JM. Subcortical control of the default mode network: Role of the basal forebrain and implications for neuropsychiatric disorders. Brain Res Bull. 2022;185:129–39. 10.1016/j.brainresbull.2022.05.005 PMC9290753 35562013

[70] Medoff DR, Holcomb HH, Lahti AC, Tamminga CA. Probing the human hippocampus using rCBF: contrasts in schizophrenia. Hippocampus. 2001;11:543–50. 10.1002/hipo.1070 11732707

[71] Carlén M. What constitutes the prefrontal cortex? Science. 2017;358:478–82. 10.1126/science.aan8868 29074767

[72] Young MD, Behjati S. SoupX removes ambient RNA contamination from droplet-based single-cell RNA sequencing data. GigaScience. 2020;9:giaa151 10.1093/gigascience/giaa151 PMC7763177 33367645

[73] Wolf FA, Angerer P, Theis FJ. SCANPY: large-scale single-cell gene expression data analysis. Genome Biol. 2018;19:15 10.1186/s13059-017-1382-0 PMC5802054 29409532

[74] Wolock SL, Lopez R, Klein AM. Scrublet: computational identification of cell doublets in single-cell transcriptomic data. Cell Syst. 2019;8:281–291.e9. 10.1016/j.cels.2018.11.005 PMC6625319 30954476

[75] Kiselev VY, Yiu A, Hemberg M. scmap: projection of single-cell RNA-seq data across data sets. Nat Methods. 2018;15:359–62. 10.1038/nmeth.4644 29608555

[76] Gu Z, Eils R, Schlesner M. Complex heatmaps reveal patterns and correlations in multidimensional genomic data. Bioinformatics. 2016;32:2847–9. 10.1093/bioinformatics/btw313 27207943

[77] Kolberg L, Raudvere U, Kuzmin I, Vilo J, Peterson H. gprofiler2 - an R package for gene list functional enrichment analysis and namespace conversion toolset g:Profiler. F1000Research. 2020;9:ELIXIR–709. 10.12688/f1000research.24956.2 PMC7859841 33564394

[78] Wang JZ, Du Z, Payattakool R, Yu PS, Chen CF. A new method to measure the semantic similarity of GO terms. Bioinformatics. 2007;23:1274–81. 10.1093/bioinformatics/btm087 17344234

[79] Sayols S rrvgo: a bioconductor package for interpreting lists of gene ontology terms. MicroPublication Biol. 2023. 10.17912/micropub.biology.000811. PMC10155054 37151216

[80] Demontis D, Walters GB, Athanasiadis G, Walters R, Therrien K, Nielsen TT, et al. Genome-wide analyses of ADHD identify 27 risk loci, refine the genetic architecture and implicate several cognitive domains. Nat Genet. 2023;55:198–208. 10.1038/s41588-022-01285-8 PMC10914347 36702997

[81] Grove J, Ripke S, Als TD, Mattheisen M, Walters RK, Won H, et al. Identification of common genetic risk variants for autism spectrum disorder. Nat Genet. 2019;51:431–44. 10.1038/s41588-019-0344-8 PMC6454898 30804558

[82] Huang QQ, Wigdor EM, Malawsky DS, Campbell P, Samocha KE, Chundru VK, et al. Examining the role of common variants in rare neurodevelopmental conditions. Nature. 2024;636:404–11. 10.1038/s41586-024-08217-y PMC11634775 39567701

[83] Savage JE, Jansen PR, Stringer S, Watanabe K, Bryois J, de Leeuw CA, et al. Genome-wide association meta-analysis in 269,867 individuals identifies new genetic and functional links to intelligence. Nat Genet. 2018;50:912–9. 10.1038/s41588-018-0152-6 PMC6411041 29942086

[84] 1000 Genomes Project Consortium, Auton A, Brooks LD, Durbin RM, Garrison EP, Kang HM, et al. A global reference for human genetic variation. Nature. 2015;526:68–74. 10.1038/nature15393 PMC4750478 26432245

[85] Finucane HK, Reshef YA, Anttila V, Slowikowski K, Gusev A, Byrnes A, et al. Heritability enrichment of specifically expressed genes identifies disease-relevant tissues and cell types. Nat Genet. 2018;50:621–9. 10.1038/s41588-018-0081-4 PMC5896795 29632380

[86] Wang M, Zhao Y, Zhang B. Efficient test and visualization of multi-set intersections. Sci Rep. 2015;5:16923 10.1038/srep16923 PMC4658477 26603754

