# Supplementary material for: Developmentally dynamic, non-convergent transcriptomic profiles in CNV models for schizophrenia risk
Source: Mol Psychiatry. 2026 Jun 18;31(11):6255–66. doi: 10.1038/s41380-026-03679-0 (PMC13668713; doi:10.1038/s41380-026-03679-0)
Supplement: Supplementary file 1 [file 41380_2026_3679_MOESM1_ESM.pdf]

Supplementary Materials

Supplementary Notes

Note 1. Our experimental design initially involved pairing two mice of different genotypes and different sexes. This approach allowed us to determine the genotype in the downstream analysis based on sex classification. Subsequently, we modified our methodology by pairing two mice of the same genotype but different sexes for the 22q11.2 mouse line and some samples from the 1q21.1 mouse line. This adjustment was made to address concerns about the accuracy and reliability of genotype determination based on sex differentiation. Through rigorous testing, we found that genotype determination was feasible using the first pairing technique, and several methods of sex classification on single cells have since been developed (ex. the classifySex function from the speckle R package). Our analysis suggests that the initial approach of pairing different genotypes with different sexes may offer a more robust method of addressing batch effects due to sample processing and library preparation. We found that samples that were processed together tended to have more similar transcriptomic profiles and that this effect was stronger than the effect of genotype on the separation of samples. This suggests that much of the transcriptomic signal that is captured by scRNA-seq is heavily influenced by the isolation of single cells and experimental workflow, highlighting the need for proper experimental controls to reduce the effect of technical artifacts.

Supplementary Figures

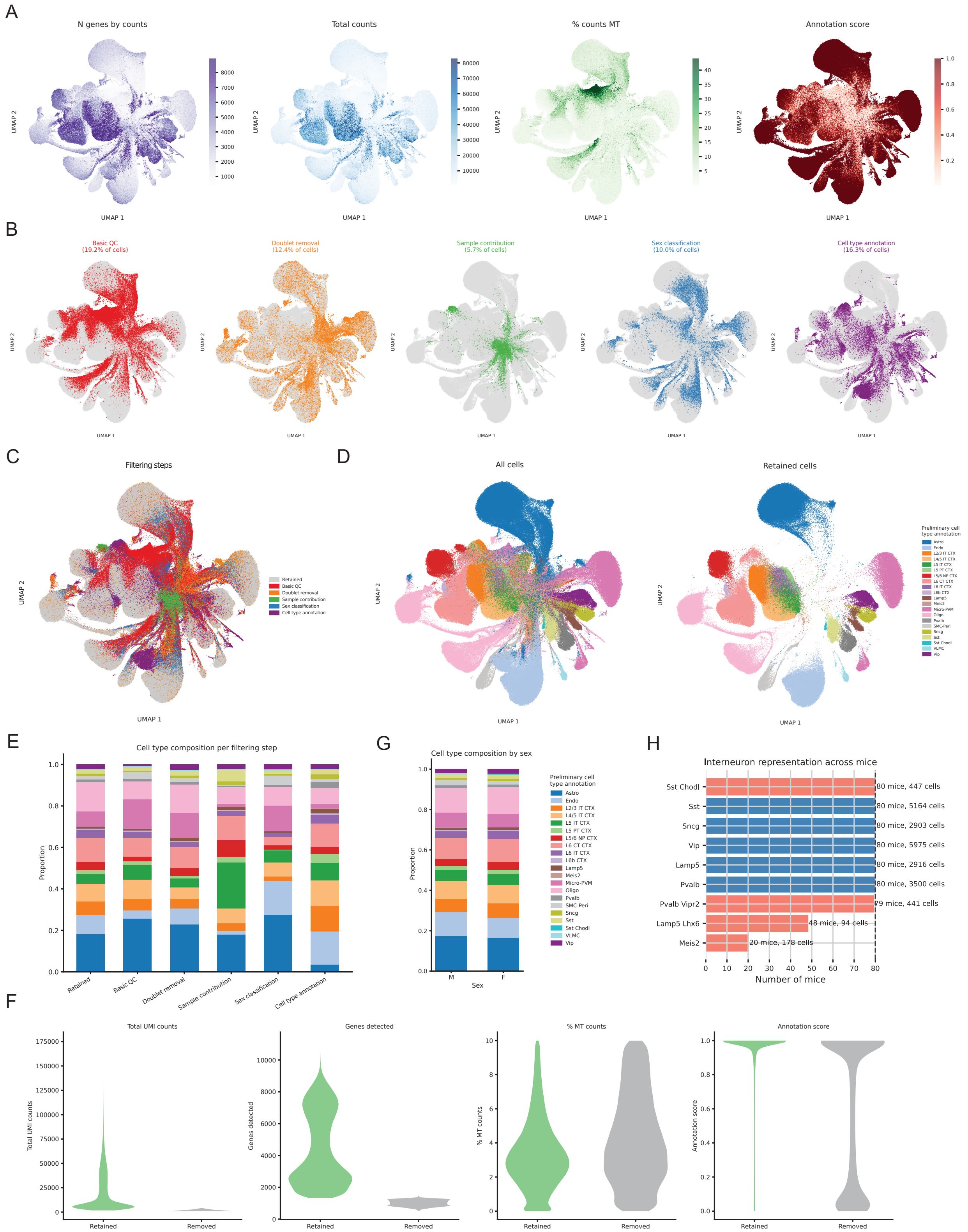

Supplementary Figure 1. A. Quality metrics visualized on UMAP representations, including number of genes per cell, total counts, percentage of mitochondrial counts, and preliminary annotation confidence

score (1 = high confidence, 0 = low confidence). **B.** UMAP representations highlighting cells removed at each filtering step. **C.** All filtering steps combined on a single UMAP, coloured by step. **D.** Preliminary cell type annotations before (left) and after (right) filtering. **E.** Preliminary cell type composition of cells retained or removed at each filtering step. **F.** Quality metrics as in **A**, highlighting cells retained or removed during the sex classification step. **G.** Preliminary cell type composition per sex group. **H.** Interneuron representation across mice. Meis2 cells were identified in the dataset but excluded due to insufficient representation across animals (present in only 20 of 80 mice). Sst-Chodl, Pvalb-Vipr2, and Lamp5-Lhx6 cells were merged with their parent clusters (Sst, Pvalb, and Lamp5, respectively) to maximise statistical power for downstream analyses.

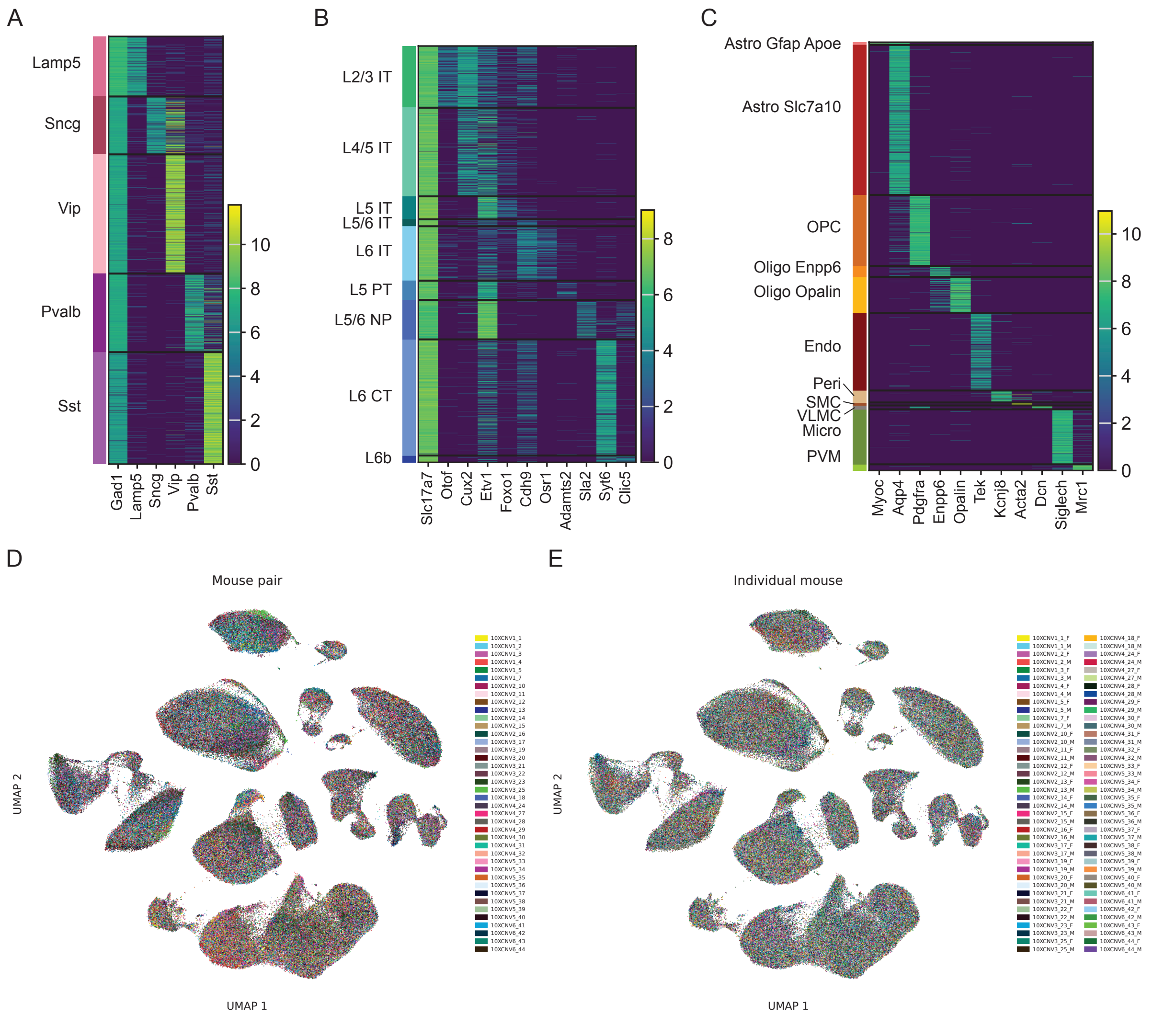

Supplementary Figure 2. A-C. Marker genes used to confirm scmap cell2cluster annotations for GABAergic (**A**), glutamatergic (**B**), and non-neuronal (**C**) cell types. **D-E**. UMAP representations coloured by mouse pair (**D**) and individual mouse (**E**), demonstrating broad representation of mice across cell clusters and absence of batch effects.

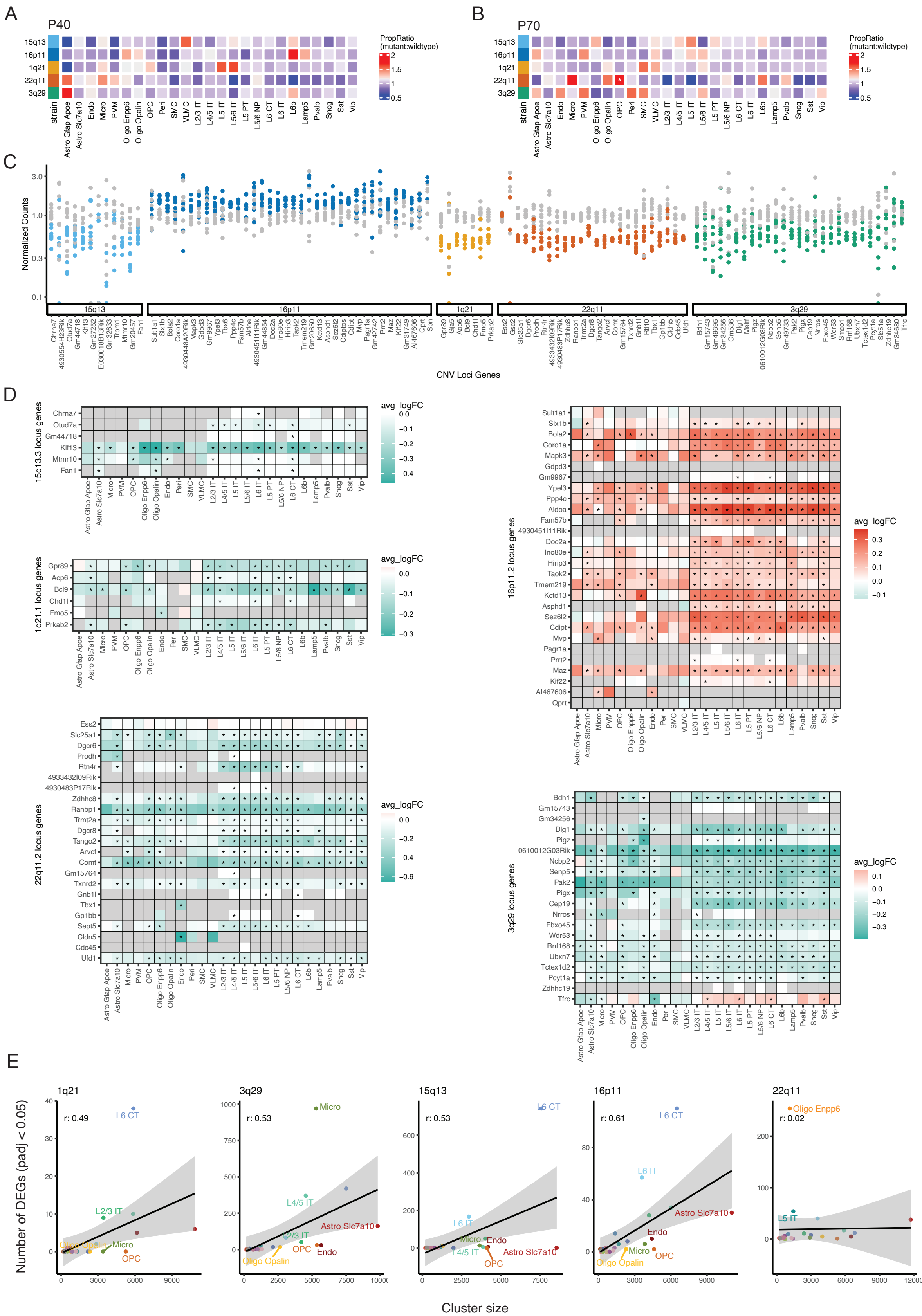

A
P40
B
P70
PropRatio (mutant:wildtype)
15q13
16p11
1q21
22q11
3q29
strain
C
Normalized Counts
CNV Loci Genes
D
15q13.3 locus genes
1q21.1 locus genes
22q11.2 locus genes
16p11.2 locus genes
3q29 locus genes
avg_logFC
E
Number of DEGs (padj < 0.05)
Cluster size

Supplementary Figure 3. A-B. Ratio of cell type proportions between mutant and wildtype mice at age P40 (**A**) and P70 (**B**), grouped by CNV model. Red indicates a higher proportion in mutants, while blue indicates a higher proportion in wildtype mice. Asterisks indicate significant differences (FDR < 0.05). **C**. Normalized expression data for detected genes within the five CNV regions. Each point represents the normalized counts for one mouse and is coloured according to its genotype (grey for wildtype mice and colour for CNV mice). For each gene, the counts have been normalized to the median expression in wildtype mice. **D**. Cell type-specific expression of genes within the 5 CNV loci, coloured by under- (blue) or over-expression (red) in mutant mice. Asterisks indicate significant differential expression (FDR < 0.05). **E**. Number of cells per cluster plotted against the number of DEGs found within the cell type with Pearson correlation coefficients.

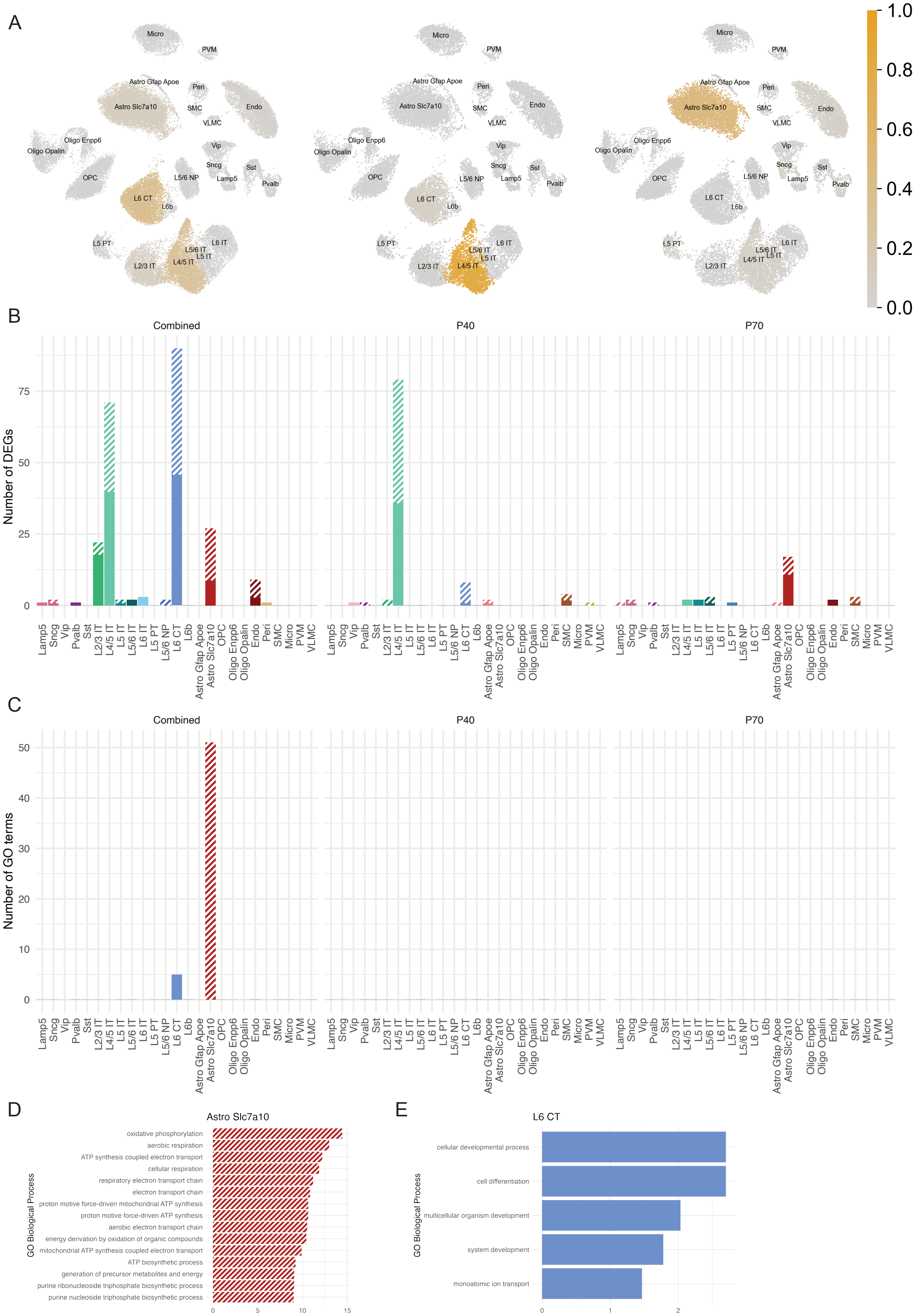

A
Micro
PVM
Astro Gfap Apoe
Peri
Astro Slc7a10
SMC
Endo
VLMC
Oligo Enpp6
Oligo Opalin
Vip
Sncg
Sst
OPC
L5/6 NP
Lamp5
Pvalb
L6 CT
L6b
L5 PT
L6 IT
L5/6 IT
L5 IT
L2/3 IT
L4/5 IT
1.0
0.8
0.6
0.4
0.2
0.0
B
Combined
P40
P70
Number of DEGs
C
Number of GO terms
Lamp5
Sncg
Vip
Pvalb
Sst
L2/3 IT
L4/5 IT
L5 IT
L5/6 IT
L6 IT
L5 PT
L5/6 NP
L6 CT
L6b
Astro Gfap Apoe
Astro Slc7a10
OPC
Oligo Enpp6
Oligo Opalin
Endo
Peri
SMC
Micro
PVM
VLMC
D
Astro Slc7a10
GO Biological Process
oxidative phosphorylation
aerobic respiration
ATP synthesis coupled electron transport
cellular respiration
respiratory electron transport chain
electron transport chain
proton motive force-driven mitochondrial ATP synthesis
proton motive force-driven ATP synthesis
aerobic electron transport chain
energy derivation by oxidation of organic compounds
mitochondrial ATP synthesis coupled electron transport
ATP biosynthetic process
generation of precursor metabolites and energy
purine ribonucleoside triphosphate biosynthetic process
purine nucleoside triphosphate biosynthetic process
-log10(p_value)
E
L6 CT
cellular developmental process
cell differentiation
multicellular organism development
system development
monoatomic ion transport
-log10(p_value)

Supplementary Figure 4. Results from the 1q21.1del model. A. UMAP projections of the 1q21.1del dataset, coloured by the proportion of total DEGs (padj < 0.1) per cell type, excluding genes located within the CNV locus. Panels from left to right show: the combined dataset, P40 samples, and P70 samples. **B**. Downregulated (stripe) and upregulated (no stripe) DEGs found per cell type. **C**. Downregulated (stripe) and upregulated (no stripe) pathways over-represented in DEGs found per cell type. **D-E**. The top 15 downregulated (D) and upregulated (E) GO terms found in astrocytes and L6 CT neurons, respectively, in the combined dataset.

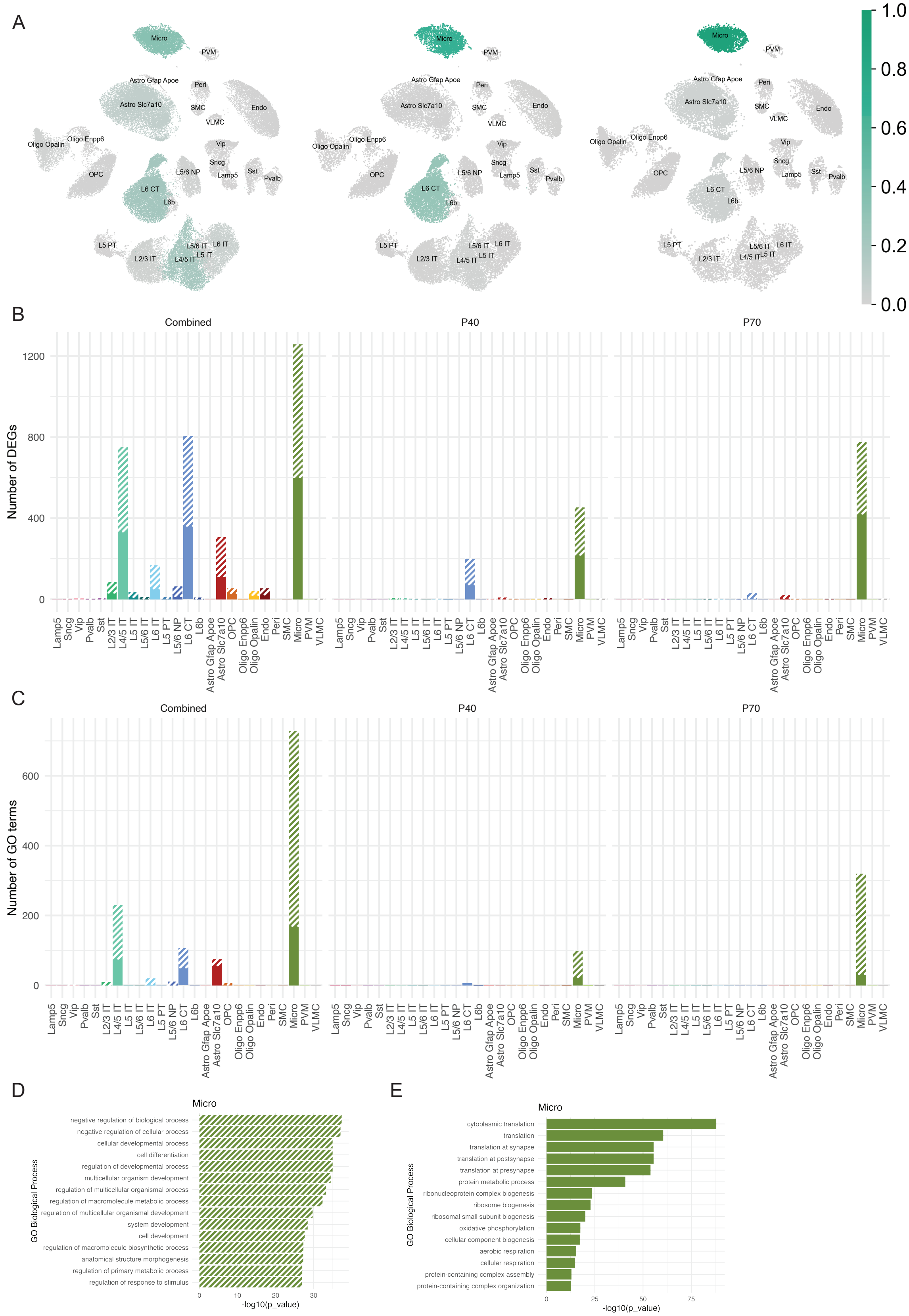
A
B
C
D
E
Micro
PVM
Astro Gfap Apoe
Peri
Astro Slc7a10
SMC
Endo
VLMC
Oligo Opalin
Oligo Enpp6
Vip
Sncg
OPC
L5/6 NP
Lamp5
Sst
Pvalb
L6 CT
L6b
L5 PT
L5/6 IT
L6 IT
L2/3 IT
L4/5 IT
L5 IT
1.0
0.8
0.6
0.4
0.2
0.0
Combined
P40
P70
Number of DEGs
Number of GO terms
GO Biological Process
-log10(p_value)
negative regulation of biological process
negative regulation of cellular process
cellular developmental process
cell differentiation
regulation of developmental process
multicellular organism development
regulation of multicellular organismal process
regulation of macromolecule metabolic process
regulation of multicellular organismal development
system development
cell development
regulation of macromolecule biosynthetic process
anatomical structure morphogenesis
regulation of primary metabolic process
regulation of response to stimulus
cytoplasmic translation
translation
translation at synapse
translation at postsynapse
translation at presynapse
protein metabolic process
ribonucleoprotein complex biogenesis
ribosome biogenesis
ribosomal small subunit biogenesis
oxidative phosphorylation
cellular component biogenesis
aerobic respiration
cellular respiration
protein-containing complex assembly
protein-containing complex organization

Supplementary Figure 5. Results from the 3q29del model. A. UMAP projections of the 3q29del dataset, coloured by the proportion of total DEGs (padj < 0.1) per cell type, excluding genes located within the CNV locus. Panels from left to right show: the combined dataset, P40 samples, and P70 samples. **B**. Downregulated (stripe) and upregulated (no stripe) DEGs found per cell type. **C**. Downregulated (stripe) and upregulated (no stripe) pathways over-represented in DEGs found per cell type. **D-E**. The top 15 downregulated (D) and upregulated (E) GO terms found in microglia in the combined dataset.

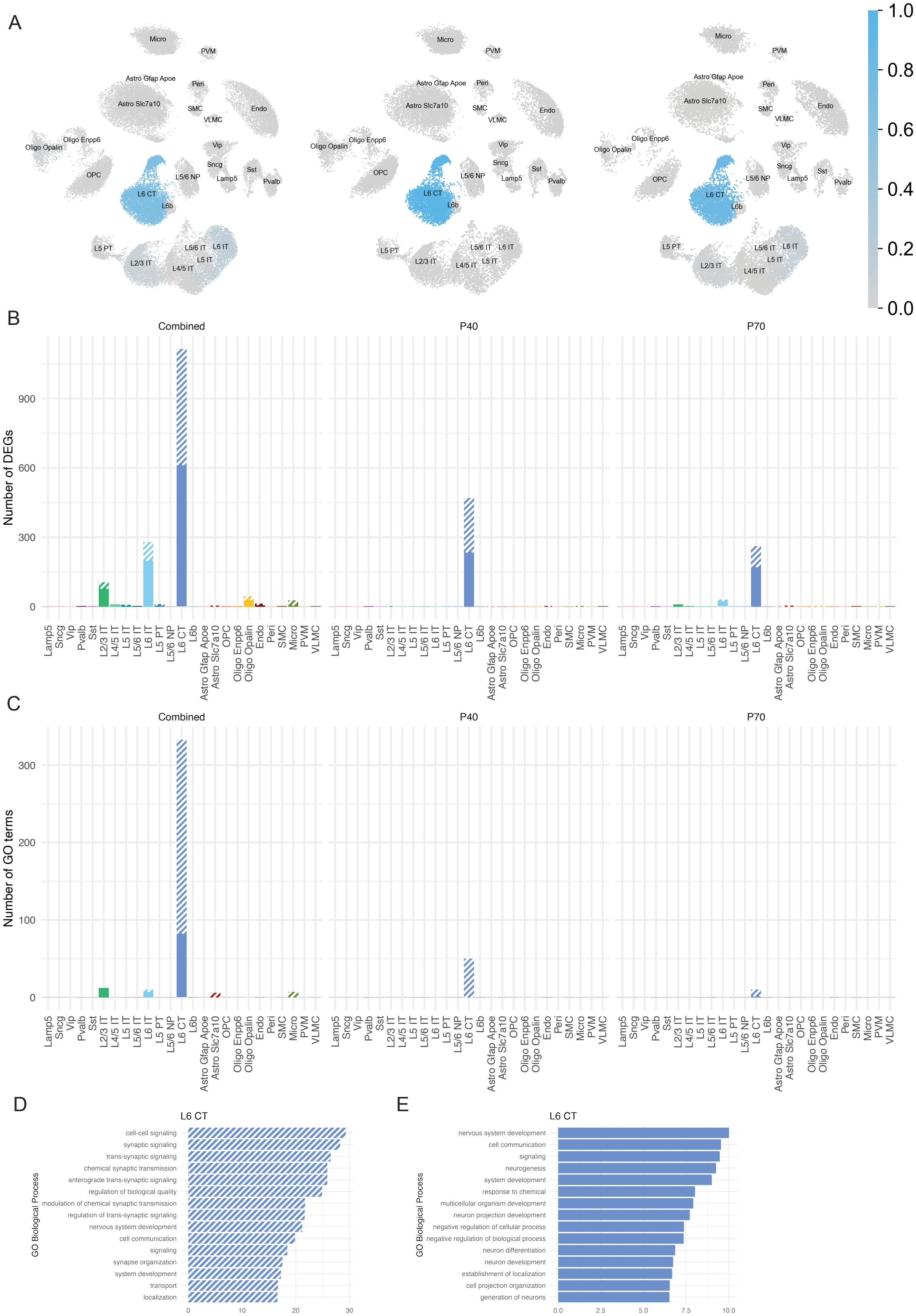
A
Micro
PVM
Astro Gfap Apoe
Peri
Astro Slc7a10
SMC
Endo
VLMC
Oligo Enpp6
Oligo Opalin
Vip
Sncg
OPC
L5/6 NP
Lamp5
Sst
Pvalb
L6 CT
L6b
L5 PT
L5/6 IT
L6 IT
L5 IT
L2/3 IT
L4/5 IT
1.0
0.8
0.6
0.4
0.2
0.0
B
Combined
P40
P70
Number of DEGs
900
600
300
0
Lamp5
Sncg
Vip
Pvalb
Sst
L2/3 IT
L4/5 IT
L5 IT
L5/6 IT
L6 IT
L5 PT
L5/6 NP
L6 CT
L6b
Astro Gfap Apoe
Astro Slc7a10
OPC
Oligo Enpp6
Oligo Opalin
Endo
Peri
SMC
Micro
PVM
VLMC
C
Number of GO terms
300
200
100
0
D
L6 CT
GO Biological Process
cell-cell signaling
synaptic signaling
trans-synaptic signaling
chemical synaptic transmission
anterograde trans-synaptic signaling
regulation of biological quality
modulation of chemical synaptic transmission
regulation of trans-synaptic signaling
nervous system development
cell communication
signaling
synapse organization
system development
transport
localization
0
10
20
30
-log10(p_value)
E
L6 CT
GO Biological Process
nervous system development
cell communication
signaling
neurogenesis
system development
response to chemical
multicellular organism development
neuron projection development
negative regulation of cellular process
negative regulation of biological process
neuron differentiation
neuron development
establishment of localization
cell projection organization
generation of neurons
0.0
2.5
5.0
7.5
10.0
-log10(p_value)

Supplementary Figure 6. Results from the 15q13.3del model. A. UMAP projections of the 15q13.3del dataset, coloured by the proportion of total DEGs (padj < 0.1) per cell type, excluding genes located within the CNV locus. Panels from left to right show: the combined dataset, P40 samples, and P70 samples. **B**. Downregulated (stripe) and upregulated (no stripe) DEGs found per cell type. **C**. Downregulated (stripe) and upregulated (no stripe) pathways over-represented in DEGs found per cell type. **D-E**. The top 15 downregulated (D) and upregulated (E) GO terms found in L6 CT neurons in the combined dataset.

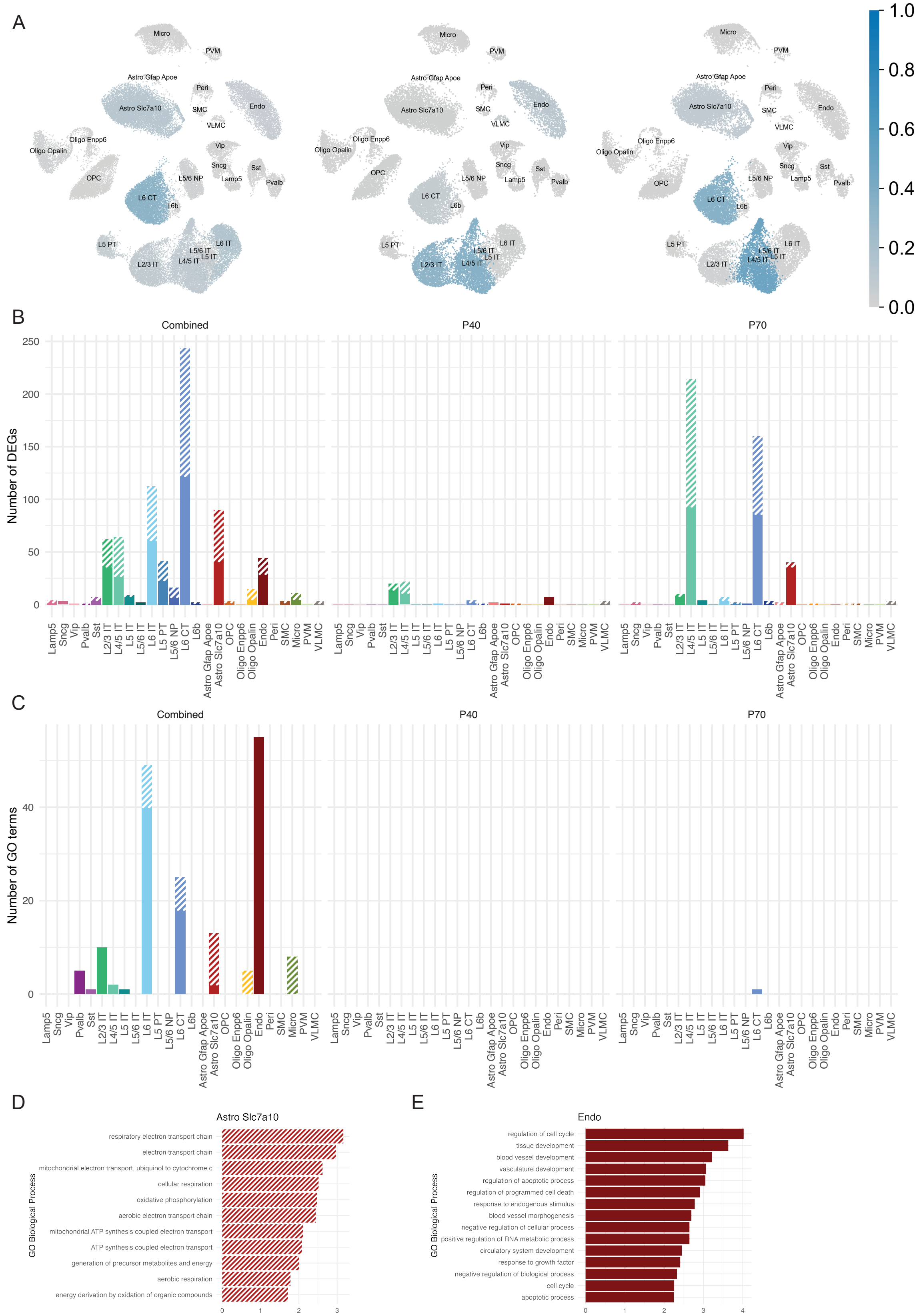
A
Micro
PVM
Astro Gfap Apoe
Peri
Astro Slc7a10
SMC
Endo
VLMC
Oligo Enpp6
Oligo Opalin
Vip
OPC
Sncg
Sst
L5/6 NP
Lamp5
Pvalb
L6 CT
L6b
L5 PT
L6 IT
L5/6 IT
L5 IT
L2/3 IT
L4/5 IT
1.0
0.8
0.6
0.4
0.2
0.0
B
Combined
P40
P70
Number of DEGs
250
200
150
100
50
0
Lamp5
Sncg
Vip
Pvalb
Sst
L2/3 IT
L4/5 IT
L5 IT
L5/6 IT
L6 IT
L5 PT
L5/6 NP
L6 CT
L6b
Astro Gfap Apoe
Astro Slc7a10
OPC
Oligo Enpp6
Oligo Opalin
Endo
Peri
SMC
Micro
PVM
VLMC
C
Combined
P40
P70
Number of GO terms
40
20
0
D
Astro Slc7a10
GO Biological Process
respiratory electron transport chain
electron transport chain
mitochondrial electron transport, ubiquinol to cytochrome c
cellular respiration
oxidative phosphorylation
aerobic electron transport chain
mitochondrial ATP synthesis coupled electron transport
ATP synthesis coupled electron transport
generation of precursor metabolites and energy
aerobic respiration
energy derivation by oxidation of organic compounds
0
1
2
3
-log10(p_value)
E
Endo
GO Biological Process
regulation of cell cycle
tissue development
blood vessel development
vasculature development
regulation of apoptotic process
regulation of programmed cell death
response to endogenous stimulus
blood vessel morphogenesis
negative regulation of cellular process
positive regulation of RNA metabolic process
circulatory system development
response to growth factor
negative regulation of biological process
cell cycle
apoptotic process
0
1
2
3
4
-log10(p_value)

Supplementary Figure 7. Results from the 16p11.2dup model. A. UMAP projections of the 16p11.2dup dataset, coloured by the proportion of total DEGs (padj < 0.1) per cell type, excluding genes located within the CNV locus. Panels from left to right show: the combined dataset, P40 samples, and P70 samples. **B**. Downregulated (stripe) and upregulated (no stripe) DEGs found per cell type. **C**. Downregulated (stripe) and upregulated (no stripe) pathways over-represented in DEGs found per cell type. **D-E**. The top 15 downregulated (D) and upregulated (E) GO terms found in astrocytes and endothelial cells, respectively, in the combined dataset.

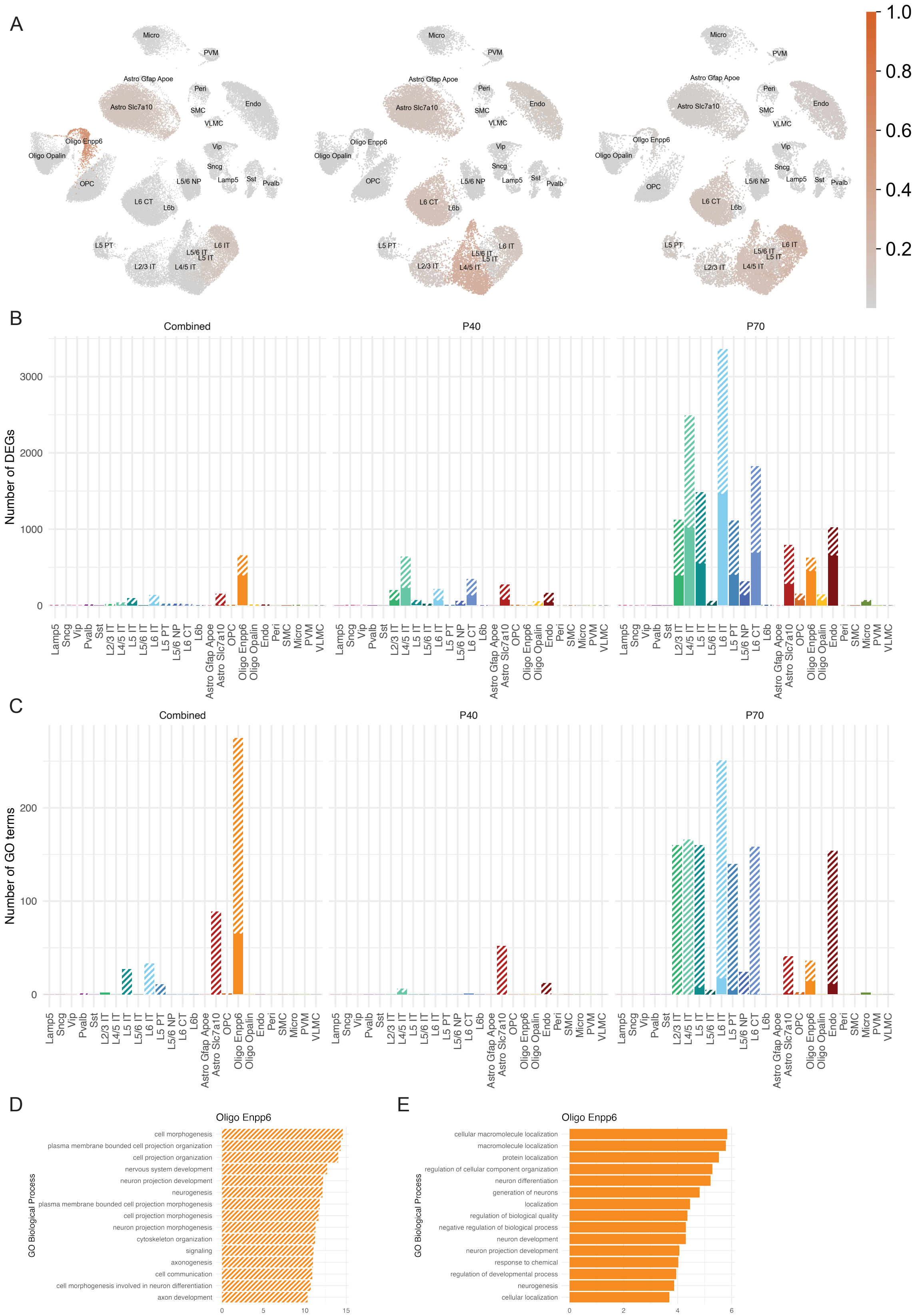

A
Micro
PVM
Astro Gfap Apoe
Peri
Endo
Astro Slc7a10
SMC
VLMC
Oligo Enpp6
Oligo Opalin
Vip
Sncg
L5/6 NP
Lamp5
Sst
Pvalb
OPC
L6 CT
L6b
L5 PT
L5/6 IT
L6 IT
L5 IT
L2/3 IT
L4/5 IT
1.0
0.8
0.6
0.4
0.2
B
Combined
P40
P70
Number of DEGs
0
1000
2000
3000
Lamp5
Sncg
Vip
Pvalb
Sst
L2/3 IT
L4/5 IT
L5 IT
L5/6 IT
L6 IT
L5 PT
L5/6 NP
L6 CT
L6b
Astro Gfap Apoe
Astro Slc7a10
OPC
Oligo Enpp6
Oligo Opalin
Endo
Peri
SMC
Micro
PVM
VLMC
C
Combined
P40
P70
Number of GO terms
0
100
200
D
Oligo Enpp6
GO Biological Process
cell morphogenesis
plasma membrane bounded cell projection organization
cell projection organization
nervous system development
neuron projection development
neurogenesis
plasma membrane bounded cell projection morphogenesis
cell projection morphogenesis
neuron projection morphogenesis
cytoskeleton organization
signaling
axonogenesis
cell communication
cell morphogenesis involved in neuron differentiation
axon development
0
5
10
15
-log10(p_value)
E
Oligo Enpp6
GO Biological Process
cellular macromolecule localization
macromolecule localization
protein localization
regulation of cellular component organization
neuron differentiation
generation of neurons
localization
regulation of biological quality
negative regulation of biological process
neuron development
neuron projection development
response to chemical
regulation of developmental process
neurogenesis
cellular localization
0
2
4
6
-log10(p_value)

Supplementary Figure 8. Results from the 22q11.2del model. A. UMAP projections of the 22q11.2del dataset, coloured by the proportion of total DEGs (padj < 0.1) per cell type, excluding genes located within the CNV locus. Panels from left to right show: the combined dataset, P40 samples, and P70 samples. **B**. Downregulated (stripe) and upregulated (no stripe) DEGs found per cell type. **C**. Downregulated (stripe) and upregulated (no stripe) pathways over-represented in DEGs found per cell type. **D-E**. The top 15 downregulated (D) and upregulated (E) GO terms found in differentiation-committed oligodendrocyte precursors (Oligo Enpp6) in the combined dataset.

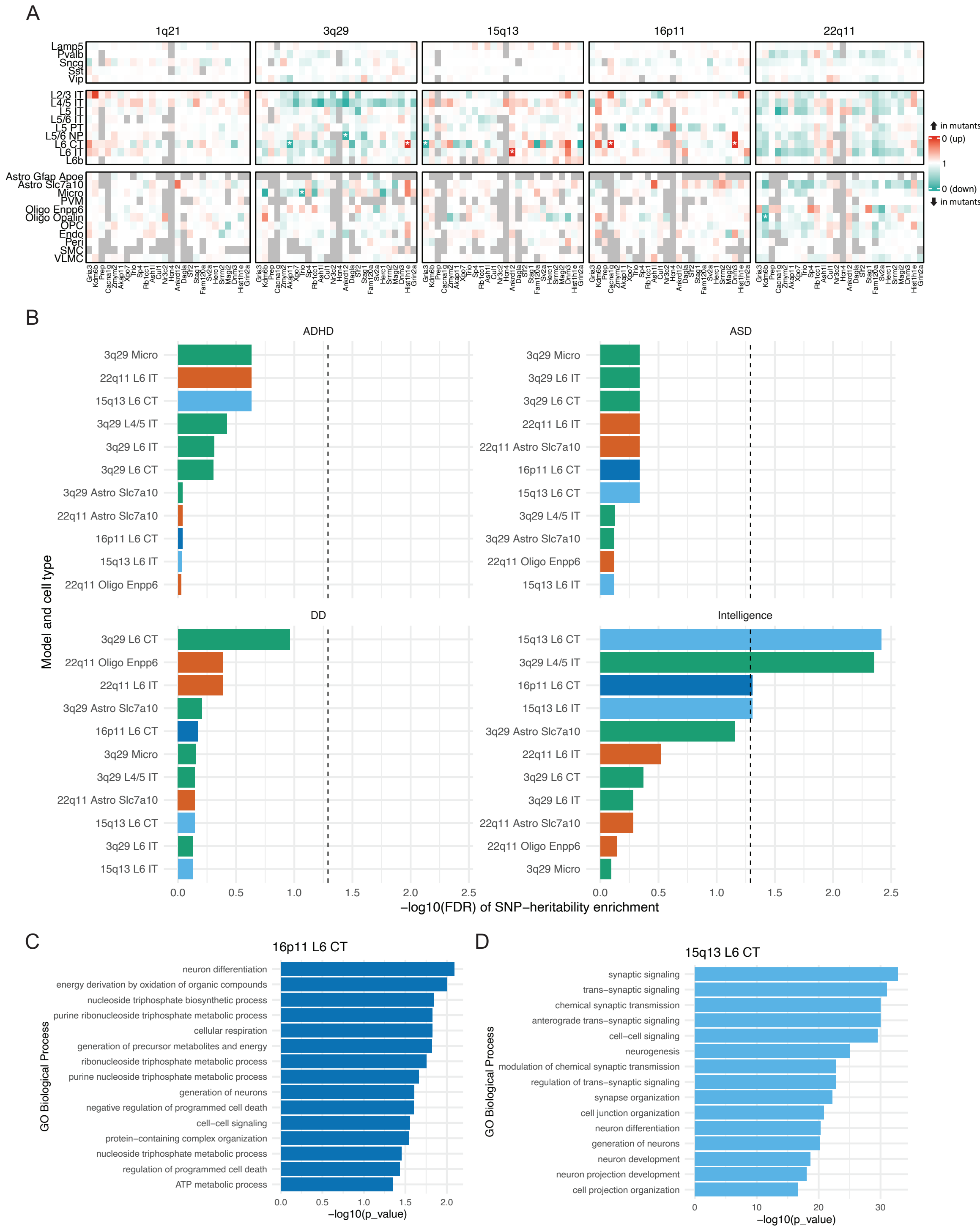

Supplementary Figure 9. A. Differential expression of rare coding variants for schizophrenia across cell types and CNV models. Color represents signed p-values and asterisks indicate significant differential expression (padj < 0.05). Grey genes were not tested due to low expression. **B**. Significance of SNP-heritability enrichment for ADHD, ASD, DD, and intelligence in DEG lists. Only DEG lists that contain sufficient numbers of SNPs (≥1%) to yield robust estimates are shown. **C-D**. The top 15 GO terms enriched in DEGs (FDR < 0.1) from 16p11.2dup (**C**) and 15q13.3del (**D**) L6 CT neurons.

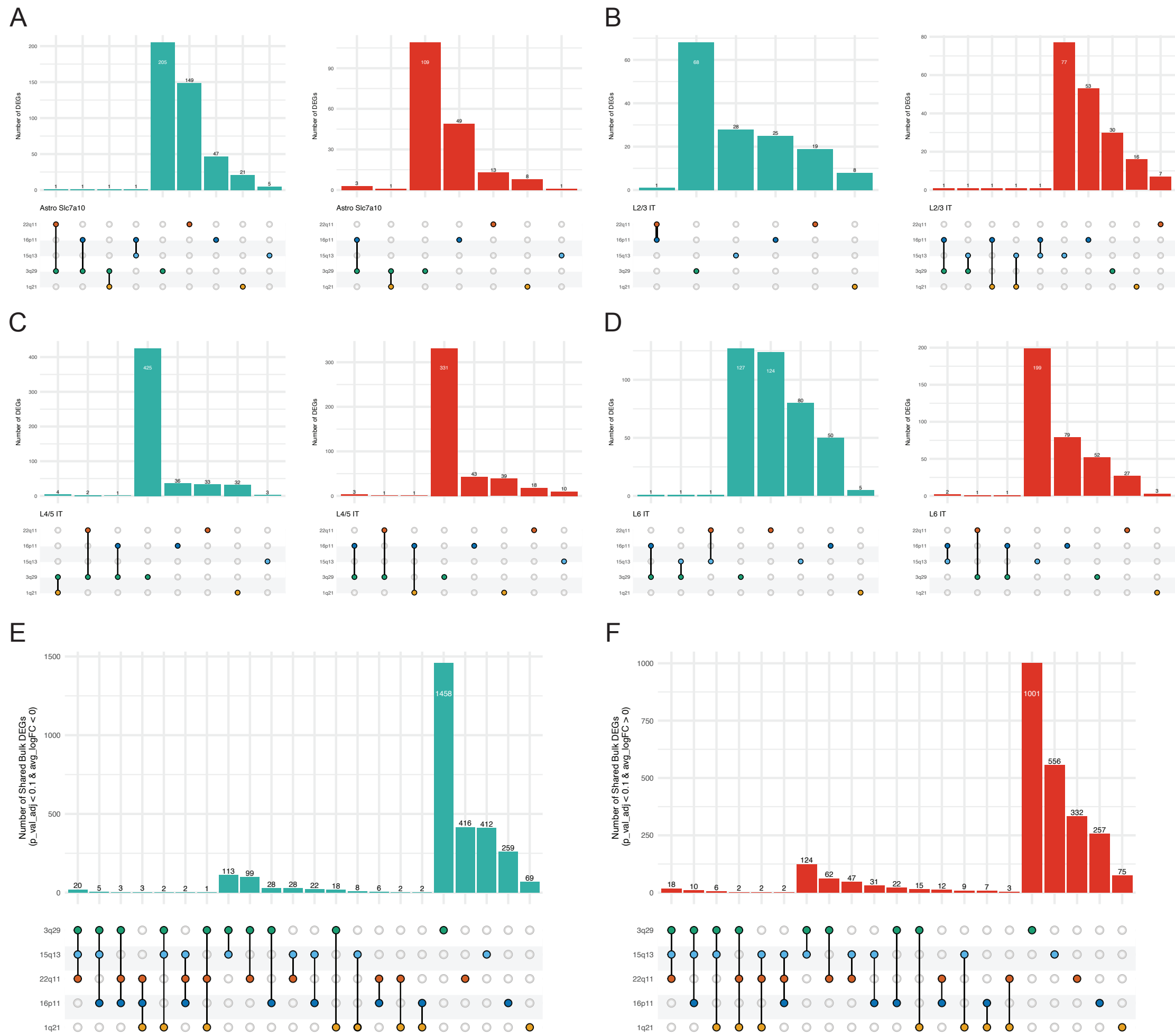

Supplementary Figure 10. A-D. Upset plots of cell types with the greatest number of downregulated (blue) or upregulated (red) shared DEGs from astrocytes (**A**), L2/3 IT neurons (**B**), L4/5 IT neurons (**C**), and L6 IT neurons (**D**). **E-F**. Upset plots of downregulated (**E**) and upregulated (**F**) DEGs on a pseudobulk level (from any cell type).

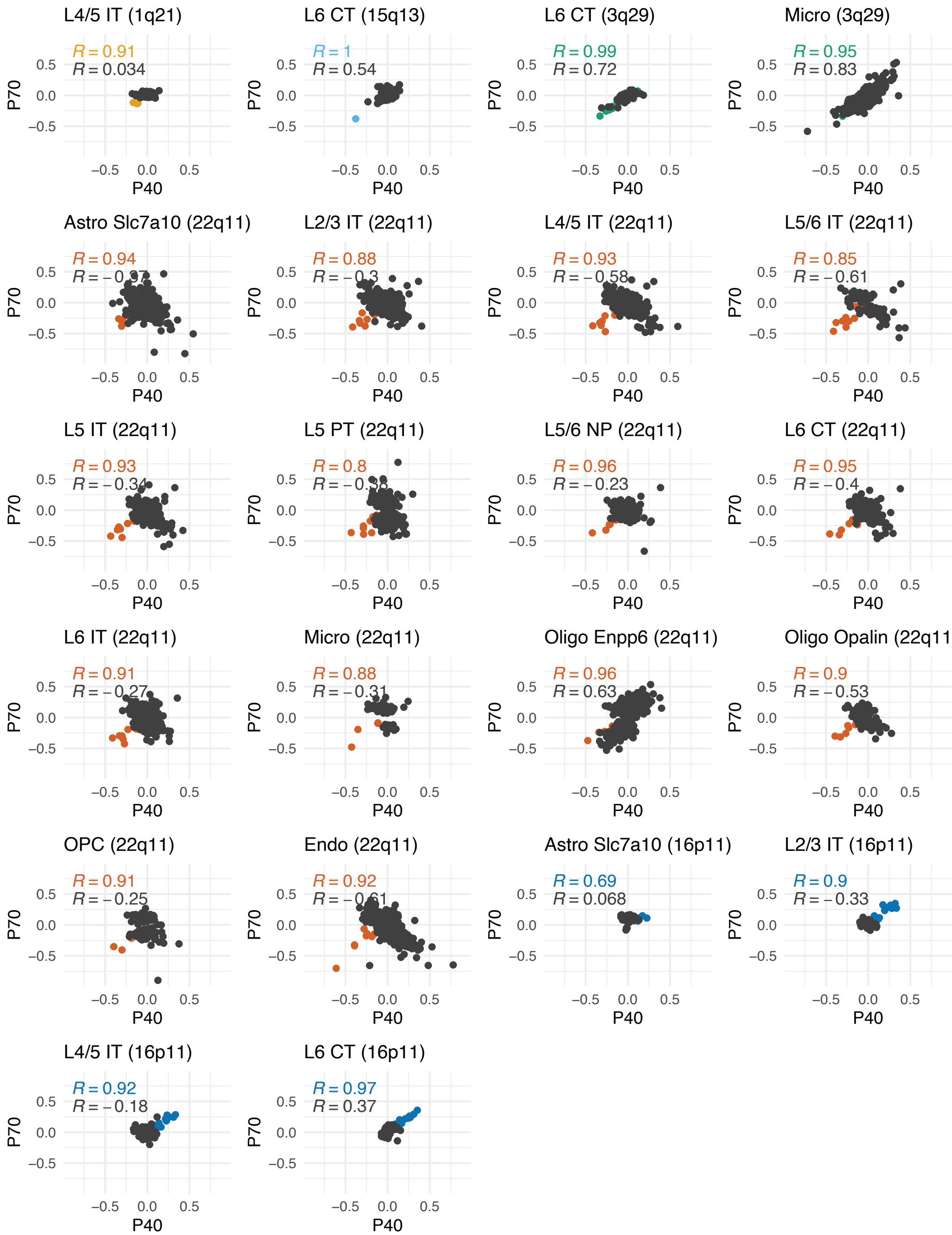

Supplementary Figure 11. Comparisons of average log2 fold change values for DEGs between age groups. Pearson correlation coefficients are calculated separately for CNV locus genes (colored) and other DEGs (black). Only cell types with more than 50 DEGs are shown.

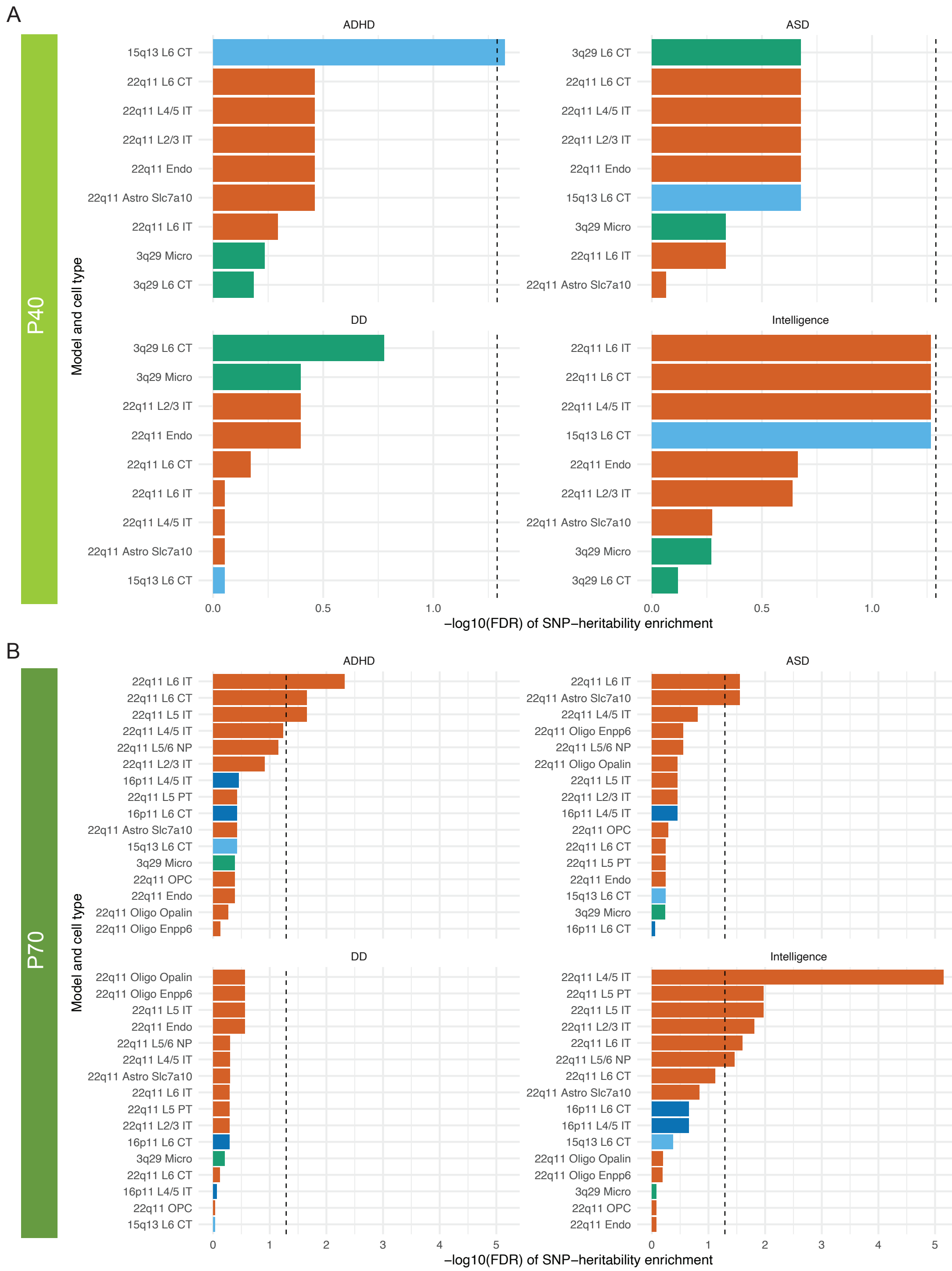

Supplementary Figure 12. A-B. Significance of SNP-heritability enrichment for ADHD, ASD, DD, and intelligence in DEG lists from P40 (**A**) and P70 (**B**) mice. Only DEG lists that contain sufficient numbers of SNPs (≥1%) to yield robust estimates are shown.

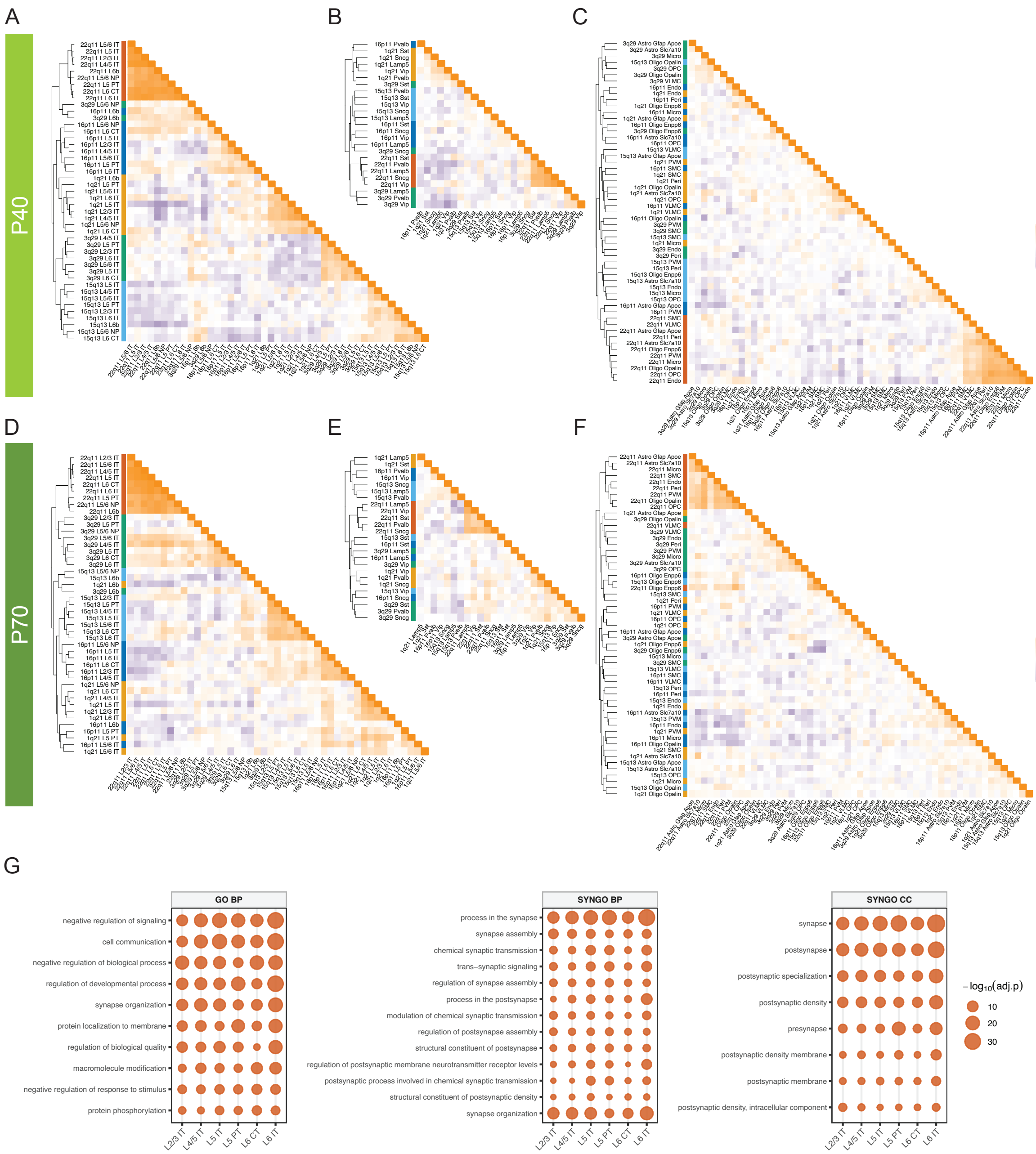

Supplementary Figure 13. A-C. Glutamatergic neurons (**A**), GABAergic neurons (**B**), and non-neuronal cells (**C**) from P40 mice. For each cell class, pairwise Pearson correlations were computed between all CNV model and cell type comparisons, based on average log2 fold change of the top 100 most differentially expressed genes per cell type (lowest adjusted p-values), followed by hierarchical clustering to assess expression concordance across conditions. **D-F.** Same analysis as in panels **A-C**, but for P70 mice. **G**. GO biological processes (left; reduced based on semantic similarity), SynGO biological processes (middle), and SynGO cellular components (right) enriched among downregulated DEGs in glutamatergic neuron types from 22q11.2del P70 mice. Only terms shared across all six cell types are shown. Dot size reflects the adjusted p-value.

A

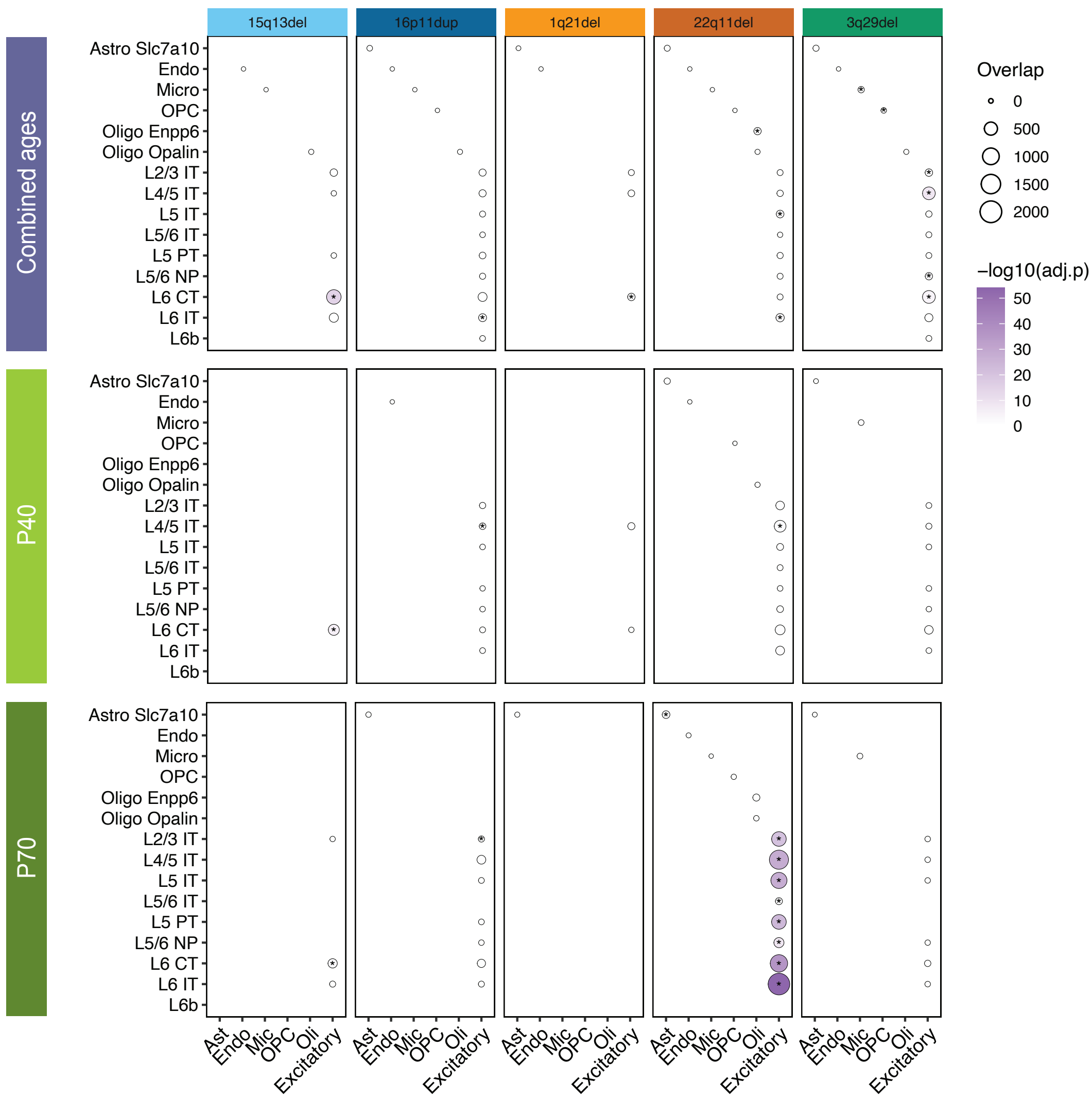

Supplementary Figure 14. A. Significance of overlap between cell type-specific DEG sets identified in each CNV model and DEGs identified by single-nuclei RNA-seq of postmortem cortical brain tissue from individuals with schizophrenia (Ruzicka et al., 2024). DEGs from all excitatory neuron subtypes in the published snRNA-seq dataset were pooled into a single gene set ("Excitatory") prior to testing to simplify the comparisons. Asterisks denote statistically significant overlaps after multiple comparison correction (p.adj < 0.05). Missing dots indicate not tested.

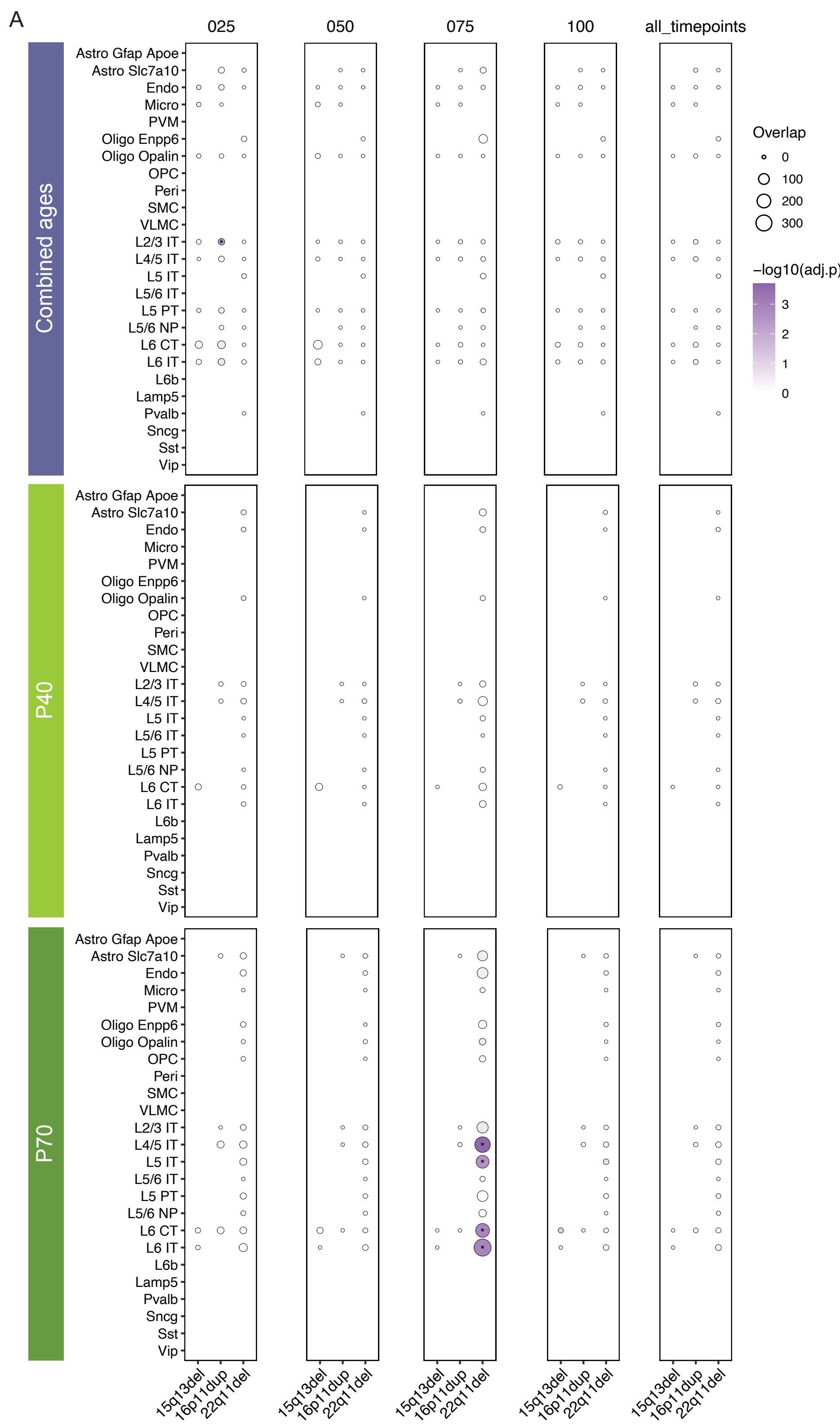

Supplementary Figure 15. A. Significance of overlap between cell type-specific DEG sets identified in each CNV model and DEGs identified by bulk RNA-seq of iPSC-derived cortical organoids from individuals with CNV syndromes (Gordon et al., 2026), excluding genes in the CNV regions. Columns indicate

differentiation day from the original study. Asterisks denote statistically significant overlaps after multiple comparison correction (p.adj < 0.05). Missing dots indicate not tested.

Supplementary Tables

Supplementary Table 1. CNV mouse models used in the study.

CNV model	CNV type	Shortform names	Mouse chromosome	Mouse gene start (bp)	Mouse gene end (bp)	Human chromosome	Human gene start (bp)	Human gene end (bp)	Provider	Mouse strain	References
15q13.3	Deletion	15q13.3del /15q13	chr7	63098692	64374095	chr15	30644082	32223648	Taconic Biosciences	10962	Fejgin et al. (2014)
16p11.2	Duplication	16p11.2dup/16p11	chr7	126672865	127137823	chr16	29584162	30181240	Thomas Arbogast via INFRAFRONTIER/EMMA	EM:06134	Arbogast et al. (2016), Qui et al. (2019)
1q21.1	Deletion	1q21.1del/ 1q21	chr3	96868281	97673812	chr1	145430995	148237104	Taconic Biosciences	11025	Nielsen et al. (2017)
22q11.2	Deletion	22q11.2del /22q11	chr16	17900709	18835261	chr22	18917768	21134566	Antonio Baldini via INFRAFRONTIER/EMMA	EM:02122	Lindsay et al. (1999), Paylor et al. (2001), Paylor et al. (2006)
3q29	Deletion	3q29del/3q29	chr16	31422280	32632794	chr3	195999964	197617792	Jennifer Gladys Mulle	B6.Del16+/Bdh1-Tfrc	Rutkowski et al. (2021)

Supplementary Table 2. Differential gene expression analysis results of CNV locus genes in all mouse models. 'Tests' indicate the total number of tested gene and cell type combinations. As expected, CNV locus genes are often differentially expressed (padj < 0.05) in their respective model and rarely differentially expressed in other models.

Locus	Compared to Locus	# Tests	# Significant Tests
15q13.3	15q13.3	72	41
15q13.3	16p11.2	85	0
15q13.3	1q21.1	76	0
15q13.3	22q11.2	89	1
15q13.3	3q29	80	0
16p11.2	15q13.3	424	0
16p11.2	16p11.2	452	281
16p11.2	1q21.1	424	0
16p11.2	22q11.2	448	1
16p11.2	3q29	435	7
1q21.1	15q13.3	89	0
1q21.1	16p11.2	90	0
1q21.1	1q21.1	83	50
1q21.1	22q11.2	96	1
1q21.1	3q29	91	0

22q11.2	15q13.3	279	1
22q11.2	16p11.2	314	2
22q11.2	1q21.1	286	0
22q11.2	22q11.2	309	216
22q11.2	3q29	294	4
3q29	15q13.3	315	1
3q29	16p11.2	333	1
3q29	1q21.1	327	0
3q29	22q11.2	357	1
3q29	3q29	330	261

Supplementary Table 3. CNV locus genes that are differentially expressed (padj < 0.05) in other models. ‘Locus’ refers to the specific locus of the gene, and ‘CNV experiment’ indicates the CNV model in which the gene was found to be differentially expressed. ‘Direction match’ indicates whether the log2(FC) value matches the under-expression of CNV locus genes from 1q21.1, 15q13.3, 3q29, and 22q11.2, or the over-expression of 16p11.2 locus genes.

**Gene name**	**Locus**	**DEG experiment**	**Cell type**	**padj**	**avg_logFC**	**Direction match**
Gpr89	1q21.1	22q11del	L4/5 IT	0.0227302133	0.03425723168	FALSE
Ubxn7	3q29	15q13del	L6 IT	0.0367441285	0.02811899041	FALSE
Tfrc	3q29	16p11dup	L4/5 IT	0.0445290021	-0.03163720276	TRUE
Dlg1	3q29	22q11del	Oligo Enpp6	0.01802184394	-0.1136874691	TRUE
Klf13	15q13.3	22q11del	Oligo Enpp6	0.01981229072	-0.1431367276	TRUE
Mapk3	16p11.2	22q11del	Oligo Enpp6	0.01458093809	-0.2171279559	FALSE
Aldoa	16p11.2	3q29del	L4/5 IT	0.005614731011	0.07839328347	TRUE
Doc2a	16p11.2	3q29del	L4/5 IT	0.01748994843	0.06314076298	TRUE
Kif22	16p11.2	3q29del	L4/5 IT	0.03143239953	-0.02873200843	FALSE
Doc2a	16p11.2	3q29del	L6 CT	0.02524246525	0.03712485226	TRUE
Bola2	16p11.2	3q29del	Micro	0.003805753843	0.08368062196	TRUE
Coro1a	16p11.2	3q29del	Micro	0.0002229565461	0.08483035197	TRUE
Aldoa	16p11.2	3q29del	Oligo Opalin	8.17E-05	0.1415746612	TRUE
Comt	22q11.2	15q13del	L6 CT	0.04087194361	-0.01600535033	TRUE
Rtn4r	22q11.2	16p11dup	L5 IT	0.03666563893	0.06941447636	FALSE
Rtn4r	22q11.2	16p11dup	L5 PT	0.01125410728	0.1109716285	FALSE
Dgcr6	22q11.2	3q29del	L4/5 IT	0.04025481354	0.03334797346	FALSE
Tango2	22q11.2	3q29del	L6 CT	0.0003671226812	-0.04613630236	TRUE
Arvcf	22q11.2	3q29del	Micro	0.001849297701	-0.05494611132	TRUE
Ranbp1	22q11.2	3q29del	Micro	0.04603352423	0.05725213457	FALSE
